# Room-Temperature Gas Sensors Under Photoactivation: From Metal Oxides to 2D Materials

**DOI:** 10.1007/s40820-020-00503-4

**Published:** 2020-08-13

**Authors:** Rahul Kumar, Xianghong Liu, Jun Zhang, Mahesh Kumar

**Affiliations:** 1 Department of Electrical Engineering, Indian Institute of Technology Jodhpur, Jodhpur, 342037 India; 2 College of Physics, Center for Marine Observation and Communications, Qingdao University, Qingdao, 266071 People’s Republic of China; 3 Key Laboratory of Advanced Energy Materials Chemistry (Ministry of Education), Nankai University, Tianjin, 300071 People’s Republic of China

**Keywords:** Gas sensor, Room temperature, Photoactivation, Metal oxide, 2D materials

## Abstract

Room-temperature gas sensors have aroused great attention in current gas sensor technology because of deemed demand of cheap, low power consumption and portable sensors for rapidly growing Internet of things applications. As an important approach, light illumination has been exploited for room-temperature operation with improving gas sensor’s attributes including sensitivity, speed and selectivity. This review provides an overview of the utilization of photoactivated nanomaterials in gas sensing field. First, recent advances in gas sensing of some exciting different nanostructures and hybrids of metal oxide semiconductors under light illumination are highlighted. Later, excellent gas sensing performance of emerging two-dimensional materials-based sensors under light illumination is discussed in details with proposed gas sensing mechanism. Originated impressive features from the interaction of photons with sensing materials are elucidated in the context of modulating sensing characteristics. Finally, the review concludes with key and constructive insights into current and future perspectives in the light-activated nanomaterials for optoelectronic gas sensor applications.

## Highlights

- Operations of metal oxide semiconductors gas sensors at room temperature under photoactivation are discussed.
- Emerging two-dimensional (2D) materials-based gas sensors under light illumination are summarized.
- The advantages and limitations of metal oxides and 2D-materials-based sensors in gas sensing at room temperature under photoactivation are highlighted.

## Graphical abstract

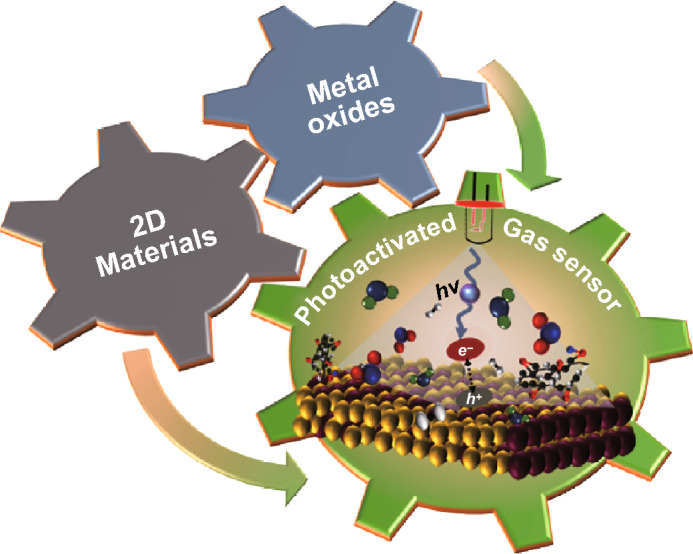

## Introduction

Over the past decades, room-temperature (RT) gas sensor device has been shown great research interest in the realm of advanced electronic devices. Due to detection of toxic gases and volatile organic compounds (VOCs), the sensors are exploited in many different kind of applications, such as air quality and industry processing monitoring, agriculture production, medical diagnosis and space [1, 2]. Various types of gas sensors such as electrochemical, optical, acoustic and conductometric, etc., have been explored in gas sensing field [3–7]. Among these sensors, resistive or field-effect transistor (FET) sensors are nowadays demanded in this nanotechnology era because of its easy fabrication, possible miniaturization, low cost and simple operation [8–12]. Moreover, different materials such as semiconducting metal oxides, carbon nanotubes (CNTs) and most emerging two-dimensional (2D) materials have been employed for developing resistive gas sensors [13–16].

Metal oxide semiconductors (MOS) have been long investigated for chemiresistive gas sensors since 1960 s [17]. This type of gas sensors usually works at an elevated temperature in the range of 200–500 °C, which requires a heater in the sensor device. Thermal energy is needed to activate the adsorption of ionized oxygen species and to overcome the barriers of sensing reactions [18–20]. However, the high working temperatures can lead to some drawbacks. It may deteriorate the working life of a sensor, increase fabrication complexity and cause decay of sensor sensitivity due to the thermally induced ripening of nanoparticles. Consequently, enormous research efforts have been dedicated to the development of gas sensors that can work at low temperature, or even RT. In this regard, light activation is a promising method as an alternative to thermal heating. The illumination of MOS with a light such as UV can change the surface electronic properties by modulating the concentration of photocarriers in MOS, hence promoting the interaction between molecules and sensing layers. It has been widely studied to improve the sensor sensitivity of various MOS at RT. In addition, light activation is also very useful to optimize the sensor selectivity and response–recovery speed. This topic has been recently discussed in some reviews and book chapters [21–23]. Here, we will summarize the most recent advances obtained in light-activated RT MOS sensors within the past few years.

On the other hand, emerging 2D materials have garnered enormous attention for developing high-performance RT chemiresistive gas sensor owing to its high surface-to-volume ratio and excellent physical or chemical properties [24–27]. First, 2D material-based chemiresistive gas sensor was fabricated using prominent 2D material graphene in [28]. The graphene gas sensor exhibited excellent sensitivity to gases even to detect single gas molecule at RT. This significant research has led exploitation of the increasing number of 2D materials in gas sensing field [24, 29–31]. Despite the RT operation with high sensitivity, slow response and incomplete recovery at RT limit its usage on commercial sensing platforms. In this regard, thermal energy was used to achieve fast response and complete recovery; however, it deteriorates the gas sensitivity of 2D material-based gas sensors [32–35]. Moreover, integration of thermal energy source with the sensor also introduces drawbacks as mentioned above for metal oxide gas sensor. On the other hand, the light source has also been utilized to address slow response/recovery kinetics of 2D materials gas sensors. Photoactivation has been improved the response/recovery time and also enhanced the gas sensitivity of the sensor at RT. Besides, it is also used for optimizing the selectivity of the 2D materials sensors. Thus, light activation is a very useful tool to optimize the sensor’s figure of merits including sensitivity, selectivity, speed and stability.

In this review, we discussed RT gas sensors using photoactivated materials. This review has been divided into two sections related to sensing materials: semiconducting metal oxide, and 2D materials including graphene and layered materials (MoS_2_, MoTe_2_, WS_2_, SnS_2_, ReS_2_, MXenes, etc.). Firstly, we focus on recent progress in gas sensing of some exciting different nanostructures and hybrids of the metal oxide semiconductors at RT under light illumination. Secondly, we discussed the gas sensing performance of emerging 2D materials under light illumination with proposed gas sensing mechanism. Finally, we explained current constructive insights and future perspective in the exploitation of photons in gas sensing field.

## Considerations of Selection of Light Source

Although light activation is an efficient method to improve the sensor performances, it is still quite difficult to tell which kind of light is most powerful towards detection of a particular molecule. This is reflected by the large amount of works reported so far, from which a general consent on the correlations between the light activation, sensor structure and materials selection is still missing.

Undoubtedly, the sensing properties are a complex interaction between sensor materials, gaseous molecules and light illumination. It is widely considered that the light illumination can change the surface carrier density of sensing materials by exciting electrons from the valence band of semiconductors. On the one hand, the bandgap of the semiconductors should be a matter of concern when choosing a light source. For example, SnO_2_ has a wide bandgap of 3.5 eV, implying this material can only be activated by light with a higher photon energy in the UV region. Probably this is why most reports of SnO_2_ sensors have been activated under UV light. In principle, TiO_2_ and ZnO materials with a moderate bandgap of ca. 3.2 eV should be active under the light illumination with a wavelength shorter than 388 nm, i.e., UV light. As reported by Kim et al. [36], the UV light (λ ≤ 382 nm) was found to result in the most significant decrease in the resistance of ZnO films due to generation of photoexcited electrons compared to the blue (λ ≤ 439 nm) and green (λ ≤ 525 nm) lights. However, this does not guarantee the best sensor response to NO, which was otherwise obtained under irradiation of blue light. It is also noted that the best sensing dynamics have been achieved under UV illumination. When PbS with a small bandgap of 0.41 eV is attached to ZnO, the sensor can be activated by near-infrared light illumination (λ = 850 nm) with a minimum photon energy or detection of NO_2_ [37]. On the other hand, the choice of light source is also related to the molecule structure. It was reported that the ZnO was not sensitive to benzene and toluene under 365 nm UV irradiation, but could be sensitive under 254 nm UV irradiation [38]. This is ascribed to the aromatic ring structure with a high stability, which needs a high photon energy to initiate the sensing reactions. Li et al. [39] showed that ZnO under UV light was very selective to formaldehyde against other molecules including methanol, acetone, toluene, benzene and ethanol. They attributed the sensitivity to the larger polarity of formaldehyde. In addition, ketone compounds change its behaviour from a weak reducing to a weak oxidizing agent under lower wavelength of 254 nm UV irradiation. So, the MoTe_2_ sensor showed different negative and positive response to ketone compounds under 365 and 254 nm light irradiation at RT, respectively [40].

In the following parts, we will present a detailed discussion on the sensor performances under photoactivation of gas sensors based on MOS, and 2D materials.

## Photoactivated Metal Oxide Semiconductors

### ZnO

ZnO nanostructures have been reported to have improved sensor sensitivity or selectivity to multiple gases under photoactivation. For gas sensors, UV illumination was initially found to largely improve the conductance of ZnO nanowires in the presence of O_2_ due to the increased carrier density, as a result of the capture of photoexcited holes by the oxygen ions (O_2_^−^, O–^−^, or O^2−^) [41]. Costello and co-workers previously demonstrated that the UV illumination successfully resulted in the RT sensitivity of ZnO thick film sensors for detection of VOCs [42]. It is rather impressive that the sensor was able to detect acetone and acetaldehyde at an extremely low concentration (1 ppb). According to this report, a tunable sensitivity of the sensor was obtained on the varied UV light intensity, and it is also possible to tune the sensor selectivity by changing the light intensity.

**Fig. 1:**
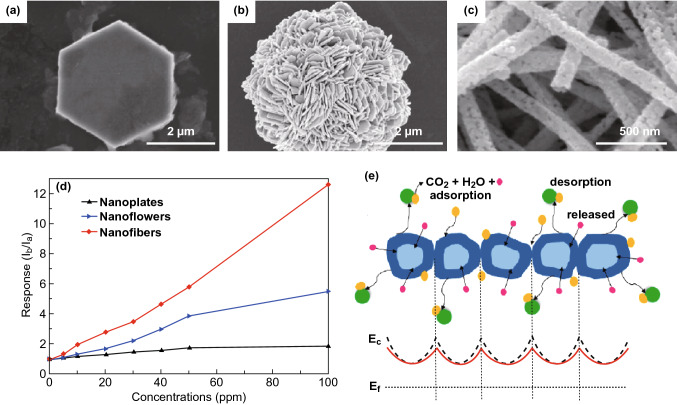
SEM images of ZnO **a** nanoplates, **b** nanoflowers, **c** nanofibers. **d** Comparative plot of sensor responses for ZnO nanoplates, nanoflowers and nanofibers at RT under UV light. **e** Schematic sensing mechanism for the ZnO nanofibers to formaldehyde under UV irradiation. Reproduced with permission [45]. Copyright 2015, Elsevier

In another work, Fan et al. studied the effects of UV illumination on the hydrogen sensing performance of ZnO thin films at RT [43]. They found that the sensor sensitivity and the response–recovery speed were improved by UV illumination. A mechanism investigation revealed that pre-chemisorbed oxygen ions (O_2_^−^) on ZnO surface are thermally stable at RT and these are unreactive in dark condition owing to its high adsorption energy. However, holes generated by UV light react with intrinsic chemisorbed oxygen ions (O_2_^-^) and desorb these from ZnO surface. While photogenerated electrons promote the additional oxygen adsorption and formation of the new highly reactive photoinduced oxygen ions (O_2_^-^), which are responsible for the RT gas sensing through performing redox reaction with target analyte at RT. Moreover, some of the gas molecules react with photoexcited electrons/holes through direct adsorption on the sensing material surface. This sensing mechanism has been widely used to explain the sensing properties of MOS under photoactivation. However, a consistent general sensing mechanism of MOS under light illumination has not been appeared yet . UV illumination was also used by Duan et al. to improve the NO_2_ sensing performances of ZnO porous thin films at RT [44]. The thickness-dependent responses were demonstrated under UV irradiation. The ZnO porous thin film with a thickness of ca. 1500 nm showed the best response compared to other thickness. They claimed the thickness-dependent responses were due to the gradual decrease of photogenerated carrier concentration in the film, which is highly related to the penetration depth of the incident UV light. This finding is meaningful to the design of sensing layers with appropriate thickness in order to achieve a high response. Furthermore, Cui and co-workers studied the effect of structural properties of ZnO on gas sensing under UV light illumination [45]. They synthesized ZnO nanofibers by electrospinning, and nanoplates as well as nanoflowers of ZnO were synthesized by hydrothermal method, and SEM images are shown in Fig. 1a–c. It was observed that ZnO nanofibers exhibited about 6.7 times higher sensitivity to formaldehyde compared to ZnO nanoplates and about 2.5 times higher than that of ZnO nanoflowers, respectively, under 365 nm UV light (Fig. 1d). This enhanced sensitivity of ZnO nanofibers was attributed to their more reactive sites on surface and polycrystalline structure with large number of grain boundaries and sensing mechanism is shown Fig. 1e. In addition, Peng et al. demonstrated sensing behaviour of ZnO nanorods to formaldehyde under UV illumination at RT [46]. The ZnO nanorods showed about 120 times higher sensitivity under UV light compared to that without UV light illumination.

A reliable selectivity to formaldehyde with low detection limit of 1.8 ppm was because of better photocatalytic oxidation of formaldehyde through absorbed oxygen ions on nanorods surface. The photocatalytic reaction is stimulated by photogenerated charge carrier efficiency; however, this efficiency decreases with decrease in size of sensing material. So, optimized size of ZnO nanorods with higher surface-to-volume ratio as well as maximum photogenerated carrier efficiency showed high sensitivity to formaldehyde at RT under UV light illumination [47]. To further enhance the photogenerated charge carrier efficiency by forming heterojunctions in sensing material, Li et al. demonstrated sensing characteristics of SnO_2_/ZnO nanofibers heterojunctions under UV light irradiation at RT [39]. The nanofibers heterojunctions increase carrier lifetime of photogenerated electron–hole pairs via avoiding recombination, which enhances the redox reaction during sensing. As a result, the SnO_2_/ZnO sensor exhibited higher selective sensitivity to formaldehyde at RT.

Synergic interaction between noble metal catalyst and photo-UV illumination has been explored to improve the sensor sensitivity. Kumar and co-workers achieved RT sensor performances from Au-modified ZnO networks to hydrogen under UV illumination [48]. The sensor exhibited a response of ~ 21.5% to 5 ppm hydrogen, while no response was recorded without UV illumination. The RT sensor response was due to the UV photoactivation enhanced the adsorption of ionized oxygen species and the d-band electron transition from Au to ZnO. UV light-activated flexible gas sensor based on ZnO materials has been reported [49, 50]. For example, nanoarrays of Au-modified ZnO nanorods have been shown by Joshi et al to have stable and reproducible performances for detection of O_3_ under UV illumination [50]. The ZnO nanorods arrays were hydrothermally grown on a poly (ethylene terephthalate) substrate to fabricate a flexible sensor. The ZnO sensor is not able to recover to its baseline resistance. The UV illumination plays a crucial role in promoting the sensor recovery. The complete recovery observed under UV irradiation is due to the accelerated reaction rate because UV light can provide sufficient energy to desorb the chemisorbed oxygen species on ZnO surfaces. Due to the formation of a nano-Schottky barrier at the Au/ZnO interface and the catalytic spillover effect of Au, the flexible Au/ZnO exhibited a high response of 108 to 30 ppb under UV illumination, which is much higher than that of ZnO. The depletion layer formed on the surface of ZnO increases the electrical resistance of the sensor. When the sensor is illuminated by UV light, many electron–hole pairs are generated because the photon energy is higher than the bandgap of ZnO. The reactions of photogenerated holes with oxygen species (O_2_^−^) will desorb the oxygen species from the ZnO surface, and the surface depletion layer is narrowed. Upon exposure to O_3_, the adsorption of O_3_ molecules on ZnO will consume the photogenerated electrons, thus causing the expansion of the surface depletion layer and the increase of the sensor resistance (Tables 1, 2, 3).

**Table 1:** Summary of metal oxide gas sensors to various gases under photoactivation at room temperature

Material	Gas	Sensitivity or response	Light source	Response/ recovery time	Detection limit	References
ZnO nanoparticles	Acetone	–	400 nm	–/–	1 ppb	[42]
ZnO nanoline	100 m H_2_	1.5%	365 nm	> 10 min	–	[43]
ZnO	50 ppm NO_2_	15	365 nm	–	–	[44]
ZnO nanofiber	100 ppm HCHO	12.61	365 nm	32/17 s	–	[45]
ZnO nanorods	200 ppm Formaldehyde	16.87	370 nm	14/0.5 min	1.8 ppm	[46]
SnO_2_/ZnO nanofibers	50 ppm HCHO	2.3	365 nm	–	–	[39]
ZnO	5 ppm H_2_	21.5%	365 nm	4/24 s	–	[48]
ZnO nanorod	30 ppb Ozone	44%	370 nm	–/–	30 ppb	[50]
Gold-ZnO	30 ppb Ozone	108%	370 nm	13.2/28.79 s	30 ppb	[50]
In_2_O_3_-ZnO	100 ppm HCHO	419%	460 nm	–/–	5 ppm	[51]
ZnO	10 ppm NO	14	439	–/–	1 ppm	[36]
ZnO/ Au NP	6 ppm NOx	78%	White	110/100 s	550 ppb	[53]
Au-ZnO	500 ppm Ethanol	62	White	–/–	1 ppm	[54]
ZnO-Ag nanoparticles	5 ppm NO_2_	1.545	470 nm	150/50 s	< 500 ppb	[55]
ZnO/PbS	1 ppm NO_2_	118–122%	850 nm	3/4 min	26 ppb	[37]
CdSe/ZnO	0–0.5 ppm NO_2_	0.7–0.8	535 nm	–/–	–	[56]
ZnO/In_2_O_3_	0.7 ppm NO_2_	117	365 nm	100/31 s	–	[58]
ZnO/g-C_3_N_4_	7 ppm NO_2_	44.8	460 nm	142/190 s	38 ppb	[59]
SnO_2_	NO_2_	300%	365 nm	2/4 min	–	[63]
Pd/SnO_2_	NO_2_	3.4 × 10^3^	365 nm	2.8/16 min	–	[65]
Pd/SnO_2_	5 ppm NO_2_	3000	365 nm	–/48 s	–	[66]
SnO_2_ monolayer array	5 ppm NO_2_	5	365 nm	7/25 s	0.1 ppm	[67]
ZnO-SnO_2_	20 ppb Ozone	8	325 nm	13/90 s	20 ppb	[68]
SnO_2_/ZnO	30 ppm Formaldehyde	40	365 nm	36/73 s	1.91 ppb	[69]
LaOCl-SnO_2_	250 ppm O_2_	2.25	380 nm	182/1315 s	–	[71]
TiO_2_ microsphere	5 ppm Formaldehyde	40	365 nm	40/50 s	124 ppb	[75]
TiO_2_@NGQD	100 ppm NO	31.1%	365 nm	235/285 s	–	[76]
In_2_O_3_	4 ppm NO_2_	8	400 nm	–	–	[77]
In_2_O_3_	50 ppm NO	40	365 nm	10 s/4 min	–	[78]
In_2_O_3_ nanorod	800 ppb NO_2_	14.9	365 nm	14/32 s	–	[79]
In_2_O_3_	50 ppm NO_2_	219	365 nm	89/80 s	–	[80]
WO_3_	160 ppb NO_2_	4	400 nm	20/42.5 min	–	[52]
WO_3_	400 ppb NO_2_	92	430 nm	51/60 min	–	[82]
PdO-WO_3_	40 ppm H_2_	8.02	Visible	2.1/5.8 min	5 ppm	[83]

**Table 2:** Summary of graphene-based gas sensors to various gases under photoactivation at room temperature

Material	Gas	Sensitivity or Response	Light source	Response/ Recovery Time	Detection limit	References
Graphene	100 ppm NO_2_	26%	265 nm	~ 200/1000 s	42.18 ppb	[87]
Graphene	10 ppt NO	1.4%	UV	–/–	158 ppq	[89]
Graphene	40 ppt NO_2_	1%	UV	–/–	2.06 ppt	[89]
Graphene	0.1 ppm Acetone	0.4%	UV	200/– s	–	[90]
Graphene	1 ppm NO_2_	20%	UV	600/900 s	–	[91]
Ti/graphene	400 ppm NH_3_	17.9%	Visible	2.5/2.7 min	–	[92]
Graphene/PS	45 ppb	2%	635 nm	1000/– s	0.5 ppb	[93]
Ag-RGO	250 ppb NH_3_	5.8	400–520 nm	76/84 s	100 ppt	[94]
WO_3_ nanorods/graphene	1 ppm NO_2_	61	Visible	–/–	–	[95]
Carbon nitride/rGO	10% O_2_	32	UV	38/39 s	20 ppm	[96]
RGO-CeO_2_	10 ppm NO_2_	4.5	365 nm	–/258 s	–	[97]
WO_3_@GO	0.9 ppm NO_2_	63.73%	480 nm	18.6/23.3 min	–	[98]
MoS_2_/rGO	10 ppm Formaldehyde	64%	> 420 nm	17/98 s	20 ppb	[99]
PGO/InGaN	100 ppm CO	32%	365 nm	70 s/10 min	–	[100]
rGO/ZnO/Pd	100 ppm CH_4_	19%	470 nm	74/78 s	5 ppm	[101]
Pd-WO_3_/Gr/Si	4 vol % H_2_	20%	980 nm	< 13/43 s	0.05 vol%	[102]
g-C_3_N_4_/rGO	2 ppm SO_2_	3%	365 nm	207/212 s	685 ppb	[103]
rGO/SnO_2_	5 ppm SO_2_	1.7%	365 nm	4.3/2.5 min	–	[74]
Graphene flexible	2.5 ppm NO_2_	290%	254 nm	281/30 s	300 ppt	[104]
Gr/bulk Si/Gr	50 ppm H_2_	20%	White	–/–	1 ppm	[105]

**Table 3:** Summary of 2D transition metal dichalcogenides and MXene gas sensors to various gases under photoactivation at room temperature

Material	Gas	Sensitivity or response	Light source	Response/ RECOVERY Time	Detection limit	References
MoS_2_	100 ppm NO_2_	160%	532 nm	–/–	–	[112]
MoS_2_	100 ppm NH_3_	70%	532 nm	–/–	–	[112]
MoS_2_	0.2% TEA	5%	White light	–/–	–	[113]
MoS_2_	100 ppm NO_2_	35.16%	365 nm	29/350 s	–	[114]
MoS_2_	100 ppm NO	70%	254 nm	250/550 s	–	[117]
MoS_2_	5 ppm NO_2_	9.2%	280 nm	–/32.9 s	–	[118]
3D Cone-Shaped MoS_2_	2 ppm NO	470%	365 nm	25 s/–	0.06 ppm	[119]
MoS_2_/graphene	NO_2_	3.3%	660 nm	–/–	0.1 ppb	[120]
MoS_2_-Au	2.5 ppm NO_2_	30%	365 nm	4/14 min	–	[123]
MoS_2–_-ZnO	50 ppb NO_2_	20%	UV	< 1/1 min	50 ppq	[124]
Sv-MoS_2_/ZnO	0.2 ppm NO_2_	226%	780 nm	75/111 s	0.1 ppb	[125]
MoS_2_ p–n junction	5 ppm NO_2_	8%	395 nm	150/30 s	8 ppb	[126]
n-MoS_2_/p-GaN	50 ppm NO	64.67%	367 nm	235/800 s	–	[127]
MoS_2_ flexible	400 ppb NO_2_	670%	625 nm	16/65 s	20 ppb	[129]
MoTe_2_	30 ppm NH_3_	790%	254 nm	–/–	3 ppb	[132]
MoTe_2_	1 ppm NO_2_	1300%	254 nm	5 min/120 s	123 ppt	[133]
MoTe_2_	100 ppm Acetone	55%	254 nm	180/180 s	200 ppb	[40]
WS_2_ nanoflakes	NH_3_	–	633 nm	20 ms/–	–	[135]
WS_2_	10 ppm NH_3_	3.4	365 nm	252/648 s	–	[136]
Au-WS_2_	250 ppb NO_2_	20%	530 nm	–/–	250 ppb	[137]
WS_2_-rGO	1 ppm NO_2_	1.27	430 nm	16/18 min	400 ppb	[138]
SnS_2_	8 ppm NO_2_	10.8	520–550 nm	164/236 s	38 ppb	[143]
SnS_2_ suspended	5 ppm NH_3_	0.34	White	300/– s	20 ppb	[145]
SnS_2_	5 ppm NO_2_	0.34	405 nm	300/– s	2.5 ppb	[144]
SnS_2_/rGO	10 ppb NO_2_	5.86	650 nm	1.5/0.54 min	0.15 ppb	[146]
ReS_2_	NH_3_	2860% (EQE)	633 nm	70/70 ms	–	[148]
Ti_3_C_2_T_x_ (MXene)	O_2_	–	200–300 nm	130/– s	–	[152]

**Fig. 2:**
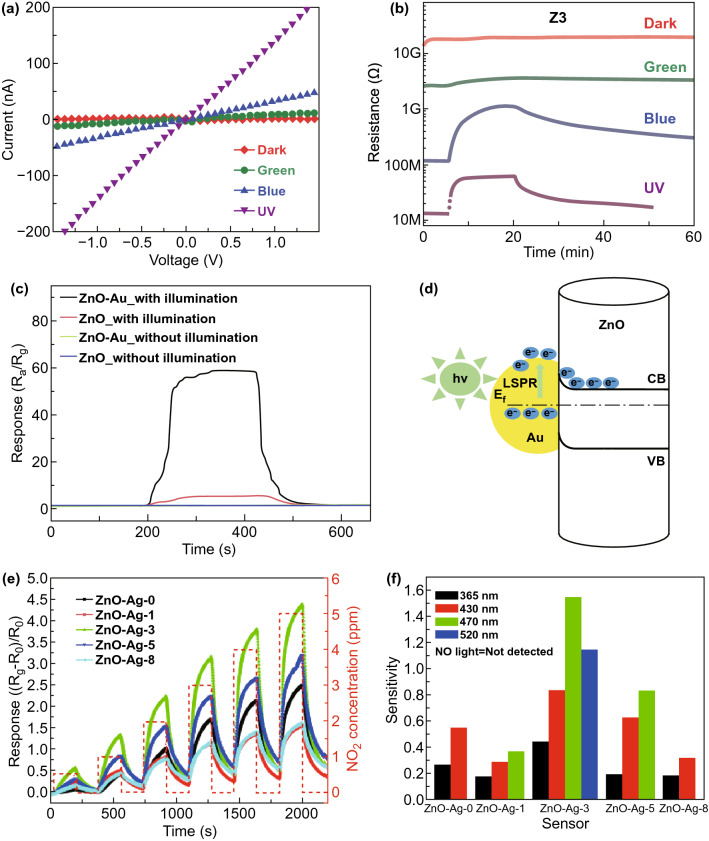
**a** Current–voltage (*I*–*V*) characteristics of ZnO film (8 nm) measured in dark and various wavelength light irradiation. **b** Resistance change of ZnO film (37 nm) to 10 ppm NO under the light of different wavelengths. Reproduced with permission [36]. Copyright 2018, Elsevier. **c** Real-time sensing response curves to 500 ppm ethanol of sensors made of ZnO and Au-decorated ZnO at RT with or without white illumination. **d** Electron transfers from Au to ZnO due to the LSPR excitations in Au. Reproduced with permission [54]. Copyright 2017, Elsevier. **e** Dynamic response curves of sensors based on pure ZnO and Ag/ZnO to 0.5–500 ppm NO_2_ under 430 nm light illumination at RT. **f** Sensitivities of sensors based on pure ZnO and ZnO-Ag heterostructure nanoparticles to 0.5–500 ppm NO_2_ illuminated at various light wavelengths at RT. Reproduced with permission [55]. Copyright 2017, Elsevier

In addition to UV, visible lights such as blue, green and red, as well as the mixed monochromatic, i.e., white light, are also frequently explored to enhance the sensor performances. The visible light activation provides higher energy efficiency and lager potential for gas sensors because of their wide spectrum range in the sunlight [51, 52]. Due to the different photon energy, the influence of different visible lights on the electronic properties and the sensor properties can be modulated. Kim and co-workers studied the *I*–*V* curves (Fig. 2a) of ZnO films in dark and various wavelength light irradiation and found that higher photon energy generated the higher current, which is due to the photoexcitation of electron–hole pairs in the film [36]. However, their gas sensing measurements revealed that the blue light irradiation exhibited the highest response (Fig. 2b), combined with Au catalytic effect greatly enhanced the NO response rate, and it is also observed that the response–recovery speed is also highly dependent on the wavelength of the lights.

Apart from the catalytic effect of noble metals, the concept of localized surface plasmon resonance (LSPR) was also utilized to develop high-performance gas sensors at RT [53, 54]. The introduction of LSPR effect into noble metal/MOS hybrids greatly expands the research in photoactivated gas sensors. Xu et al. studied the sensing performance to ethanol of Au/ZnO nanowires under white light illumination at RT [54]. They found light illumination and Au decoration jointly led to the enhanced gas sensing results. However, as shown in Fig. 2c, the Au nanoparticles are observed to play a dominant role in the enhanced sensing. They attributed the promotion effect to the LSPR effect of Au. The LSPR effect not only enhanced the light absorption but also suppress the recombination of photogenerated electron–hole pairs. The hot electrons in Au generated by the LSPR absorption can overcome the Schottky barrier at Au/ZnO junctions and inject into the conduction band of ZnO (Fig. 2d). As a result, more surface-adsorbed oxygen species will be formed on the surface of ZnO to trigger more intense sensing reactions.

Tai and co-workers investigated the sensitivities of Ag/ZnO sensors to NO_2_ gas (0.5–5 ppm) under various light (365–520 nm) illumination [55]. They also studied the loading level of Ag on the photoactivated sensor performance. The best response towards NO_2_ detection was obtained on the 3 mol% Ag/ZnO sensor under blue-green illumination with a wavelength of 470 nm (Fig. 2e). It is revealed in Fig. 2f that the varied light with different wavelength generally improves the sensor sensitivity, but this improvement is also related to the Ag loadings.

**Fig. 3:**
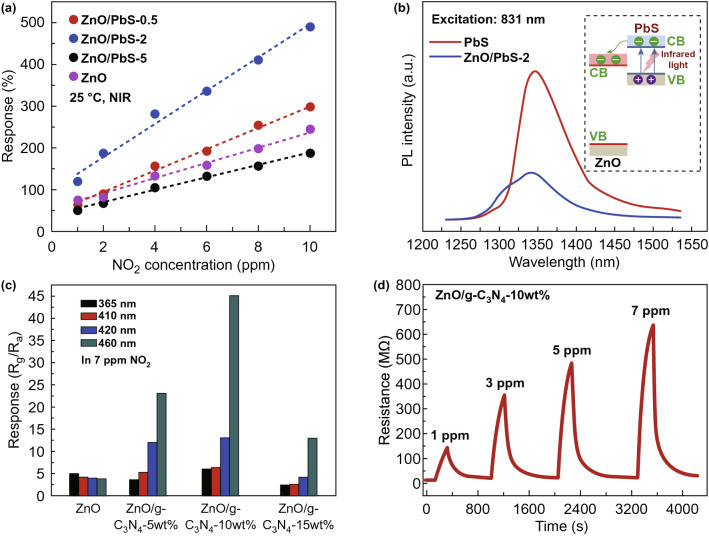
**a** Responses of the sensors based on the ZnO/PbS nanocomposites with different PbS loading to 1–10 ppm of NO_2_ under NIR illumination at RT. **b** Photoluminescence spectra of PbS and ZnO/PbS-2 under 831 nm excitation. Reproduced with permission [37]. Copyright 2017, Elsevier. **c** Responses of ZnO/g-C_3_N_4_ composites with various g-C_3_N_4_ content to 7 ppm NO_2_ under different wavelength light illumination. **d** Dynamic resistance curves of ZnO/g-C_3_N_4_-10 wt% to 1–7 ppm NO_2_ under 460 nm light illumination at RT. Reproduced with permission [64]. Copyright 2019, Elsevier

Apart from the noble metals, another photosensitizer such as quantum dots such as PdS [37] and CdSe [56] has been also functionalized on MOS to achieve better performance under photoactivation. The quantum dots are typically narrow bandgap semiconductors, e.g., 0.41 eV of PbS. When attached to MOS, the quantum dots serve as a photosensitizer to shift the optical adsorption range of MOS to higher wavelengths. On photoexcitation, the free electrons in quantum dots can migrate into the conduction band of MOS [57]. Xiang et al. studied the NO_2_ sensing performances of ZnO/PbS nanocomposites with different PbS densities near-infrared light (NIR) illumination (λ = 850 nm) [37]. As displayed in Fig. 3a, the ZnO/PbS-2 with medium PdS loading (∼ 2%) possesses the maximum response. The enhanced NO_2_ sensing performances of ZnO/PbS under NIR illumination are due to the increased carrier concentration in ZnO nanorods. The electron transfer from PbS to ZnO has been evidenced by the photoluminescence spectra (Fig. 3b) and *I*–*V* tests.

Although quantum dots of metal chalcogenides are effective in promoting the sensor performance, they suffer from high toxicity of Pd and Cd. Alternatively, the Lu group reported the use of ZnO-based composite nanomaterials for photoactivated gas sensors [58, 59. For example, they proposed the use of graphitic carbon nitride (g-C_3_N_4_) with a bandgap of 2.7 eV as the photosensitizer to enhance the ZnO sensors under the illumination of visible lights. As can be seen in Fig. 3c, the ZnO/g-C_3_N_4_-10 wt% shows the best response to NO_2_ and fast response–recovery characteristics (Fig. 3d) when activated by 460 nm visible light. It also reveals that the response of all ZnO/g-C_3_N_4_ composites to NO_2_ generally improves with the increase in wavelength.

Photoactivation of gas sensors enables the detection of gaseous molecules at RT; however, the progress discussed above generally used an external light source like Xe-lamps or LEDs. The power consumption of such devices can be down to sub-milliwatts. To fulfil the future development of the Internet of things (IOTs), miniaturized sensors with an integrated light source with an ultralow-power consumption are highly urgent. Recently, several groups have reported an appealing monolithic integration form of photoactive sensors, in which a micro-LED with a power down to microwatts is mounted with the sensing films. This kind of sensor device has some merits that are not available from the external light-activated sensors such as much lower power, more uniform irradiation of the sensor materials and higher photon energy efficiency.

**Fig. 4:**
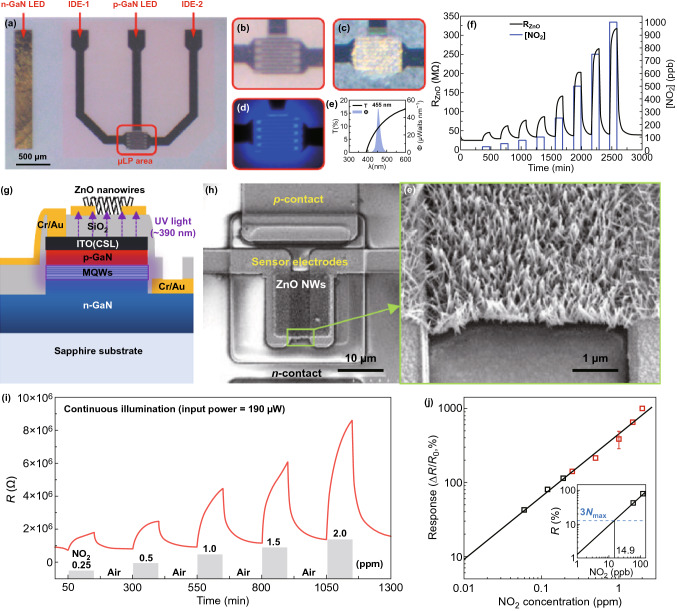
**a** Overview of the sensor device with ZnO nanoparticles on top of the micro-LED. Details of the sensor device **b** bare IDE, **c** ZnO material on top of the IDE, and **d** LED light on. **e** Light emission spectrum of the micro-LED and light transmission spectrum of the ZnO layer deposited on a sapphire substrate. **f** Resistance transient of the sensors to increasing NO_2_ concentrations. Reproduced with permission [60]. Copyright 2019, American Chemical Society. **g** Schematic cross section illustration of the photoactive sensor with ZnO nanowires grown on a micro-LED. **h** SEM images of ZnO nanowires on the micro-LED. **i** NO_2_ sensing performance of the photoactive under operating power of 190 μW and **j** calibration of normalized sensor response to NO_2_. Reproduced with permission [61]. Copyright 2020, American Chemical Society

Figure 4a–d exhibits an integrated gas sensor with ZnO nanoparticle film deposited on a micro-LED with a distance of a few hundred nanometres [60]. The sensor is activated with a visible light (emitting at 455 nm) at RT. The sensor shows a response of 20% to 25 ppb NO_2_ at an ultralow-power of 30 μW and can be improved to 94% at 200 μW. A fully recoverable detection of NO_2_ ranging from 25 ppb to 1 ppm is also shown in Fig. 4f. Park and co-workers recently reported a monolithic photoactivated gas sensor based on ZnO nanowires grown on a micro-LED, as shown in Fig. 4g, h [61]. Under the activation of UV light of 390 nm, the sensor resistance is observed to increase with the NO_2_ concentration in the range of 0.25-2 ppm at an operating power of 190 μW. The calibration of sensor response in Fig. 4j reveals a LOD of 14.9 ppb to NO_2_. Although these micro-LED integrated gas sensors have low power consumption, the sensor response dynamics in Fig. 4f, i is very slow and more efforts are need to improve the response speed.

### SnO_2_

SnO_2_ is the most widely used materials for MOS gas sensors due to its high sensitivity and good stability ever since its integration into a real sensor device by Taguchi in the 1960 s [17]. Significant efforts have been explored to lower the high working temperature by fabricating special nanostructures, synthesis of the nanocomposite and surface modification, as well as using photoexcitation instead of thermal heating.

Saura initially studied the gas sensing performance of SnO_2_ films towards acetone under UV irradiation with varying wavelengths at RT [62]. They stated that the sensor response originated from the photo-dissociation and desorption of the chemisorbed molecules. Later, Comini and co-workers investigated the NO_2_ sensing performance of SnO_2_ films [63]. They showed a stable and sensitive sensor working at RT with UV excitation (λ = 365 nm). The UV irradiation enables the fast and full recovery of the sensor by preventing the poisoning of SnO_2_ surface from strongly adsorbed NO_2_. The accelerated desorption of NO_2_ from SnO_2_ sensors by white light illumination was also observed by Anothainart et al. [64] They showed that the activated desorption was due to the light with a wavelength less than λ = 600 nm, and the light intensity also affected the desorption. By measuring the conductance and the work function at both RT and elevated temperature, they deduced the light-activated desorption was due to the direct photoexcitation of the electrons from NO_2_^-^ adsorbates into the conduction band of SnO_2_, rather than the recombination of electron–hole pairs. Recently, Hyodo et al. also reported that UV light irradiation (365 nm) enhanced the NO_2_ response of the SnO_2_ sensor at RT [65], and the response can be improved by incorporation of Pd or Pt [66].

**Fig. 5:**
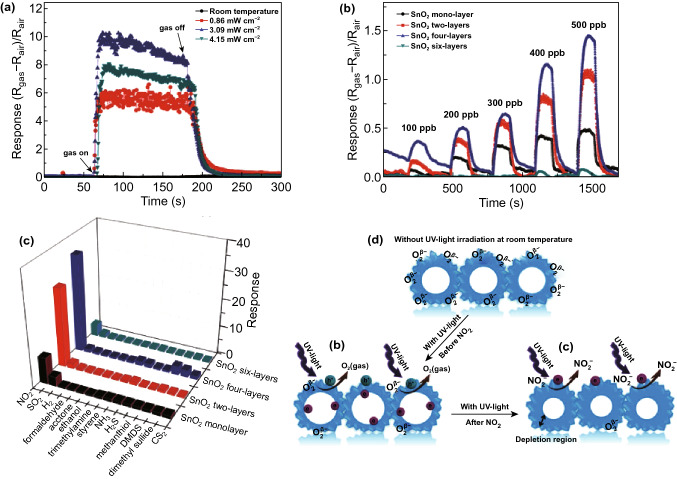
Response–recovery curves of **a** SnO_2_ monolayer array film towards 10 ppm NO_2_ under different light intensity and **b** SnO_2_ film with different thickness towards 100–500 ppb NO_2_ under the UV light intensity of 3.09 mW cm^−2^, **c** corresponding sensor responses to 10 ppm NO_2_. **d** Sensing mechanism of the SnO_2_ monolayer sensing film towards NO_2_ gas under UV light irradiation. Reproduced with permission [67]. Copyright 2019, WILEY‐VCH Verlag GmbH & Co. KGaA, Weinheim

Liu et al. recently developed an ultrasensitive NO_2_ gas sensor based on SnO_2_ monolayer array films under UV illumination [67]. The sensor response is largely affected by light intensity, as shown in Fig. 5a. They also fabricated gas sensors with different array layers of SnO_2_ nanospheres and found that the sensor with four layers exhibited the highest response (Fig. 5b) with excellent selectivity to NO_2_ against many other molecules (Fig. 5c). Sensing mechanism follows the photoactivated desorption of pre-adsorbed oxygen and subsequent adsorption of NO_2_, as depicted in Fig. 5d. On illumination, the built-in electric field in SnO_2_-induced separation of electron–hole pairs; then, the photogenerated holes react with surface-absorbed oxygen ions to give molecular O_2_. The depletion layer around the SnO_2_ spheres is reduced due to the excess of the photogenerated electron, resulting in the decreased sensor resistance. When exposed to NO_2_, the photoelectrons induced the adsorption of NO_2_ to give NO_2_^−^, resulting in an increase of electron depletion and hence the sensor resistance. Efforts have been explored to fabricate heterojunctions from semiconductors such as SnO_2_/ZnO [68–70]. The formation of heterojunctions has been proposed to suppress the recombination of photoexcited electrons and holes, thus leading to the improved performance of the UV-activated SnO_2_ gas sensor.

To further improve the photoactivated sensor performance of SnO_2_ at RT, other materials such as LaOCl [71], polypyridine Ru(II) complexes [72], perovskite methylammonium tin iodide (MASnI3) [65], perylene diimide [73] and reduced graphene oxide [74] have been incorporated with SnO_2_ to serve as a photosensitizer to widen the spectrum into visible range or as a separator to prevent the combination of photoexcited electron–hole pairs. Xue group showed that LaOCl-doped SnO_2_ hollow spheres exhibited significantly improved selective response to O_2_ under UV light illumination at RT, due to improved generation of electron–hole pairs and enhanced oxygen adsorption enabled by oxygen vacancy defect due to the presence of LaOCl dopant. Xu group reported that under UV illumination (λ = 365 nm) the sensor based on Au/MASnI_3_/SnO_2_ exhibited high response, fast recovery and good selectivity to NO_2_ compared to sensors based on SnO_2_ or Au/SnO_2_ [65]. They ascribed the enhanced sensing performance to the improved light absorption due to MASnI3, which allowed more photoelectrons transfer from MASnI_3_ to SnO_2_, as well as the catalysis of Au nanoparticles. An organic photosensitizer, i.e., heterocyclic Ru(II) complex, has been proposed by Gaskov group to shift the photosensitivity range of SnO_2_ towards visible light wavelengths [72]. The Ru(II) complex enables the sensor to have improved response to detecting NO_2_ under periodic illumination with blue (λ = 470 nm), green (λ = 535 nm) and red (λ = 630 nm) light. The sensing mechanism involves the photoexcitation of electrons from the HOMO to LUMO of Ru(II) complex and then transfer to the conduction bands of SnO_2_.

**Fig. 6:**
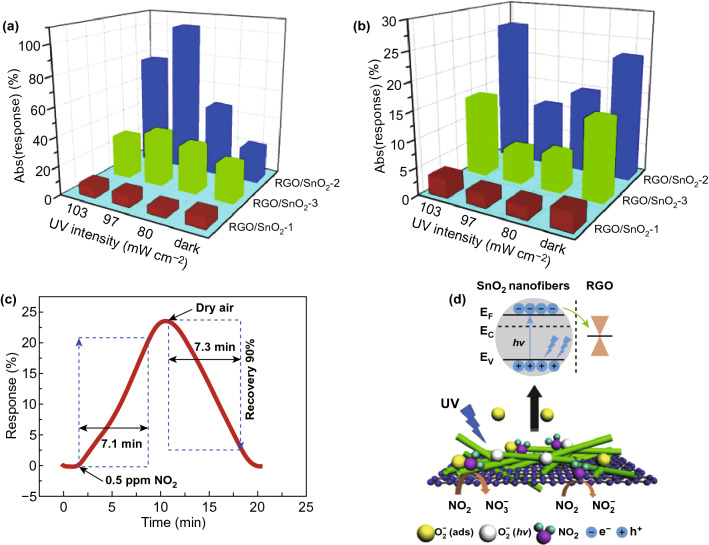
UV intensity-dependent response absolute value based on rGO/SnO_2_ sensors in **a** 3 ppm NO_2_ and **b** 30 ppm SO_2_. **c** Enlarged part of response–recovery cureves of rGO/SnO_2_ sensor to 0.5 ppm NO_2_. **d** Sensing mechanism of rGO/SnO_2_ to NO_2_ under UV illumination. Reproduced with permission [74]. Copyright 2019, Elsevier

Ren group recently realized the selective detection of NO_2_ and SO_2_ on a UV-activated gas sensor based on reduced graphene oxide (rGO)/SnO_2_ nanofiber composites at RT [74]. The improved selectivity was attributed to the combination of photocatalytic oxidation and photo-chemical desorption arising from the nanocomposite. Their results also showed that the sensor response to NO_2_ (Fig. 6a) and SO_2_ (Fig. 6b) was highly relevant to the composition ratio of rGO and SnO_2_, as well as the light intensity. The enhanced sensor responses were attributed to the synergistic effect of two materials, including prominent electron transfer, efficient material structure and p-n heterojunctions. However, this sensor suffers a very sluggish long response and recovery speed (Fig. 6c). Figure 6d shows the sensing mechanism. Under UV illumination, SnO_2_ acts as a light absorber and electron–hole pairs are generated on light excitation. The photoelectrons move to rGO, which serves as both a photoelectron acceptor and pathway for charge transport. The increased photoelectrons in rGO promoted the absorption of oxygen species and thus contribute to gas sensing reactions.

### TiO_2_

TiO_2_ has drawn paramount attention as a photocatalyst, while limited research has been paid to photoactivated gas sensors. Li et al. showed that mesoporous TiO_2_ hollow spheres exhibited high sensitivity and selectivity to formaldehyde at RT with UV illumination [75].

**Fig. 7:**
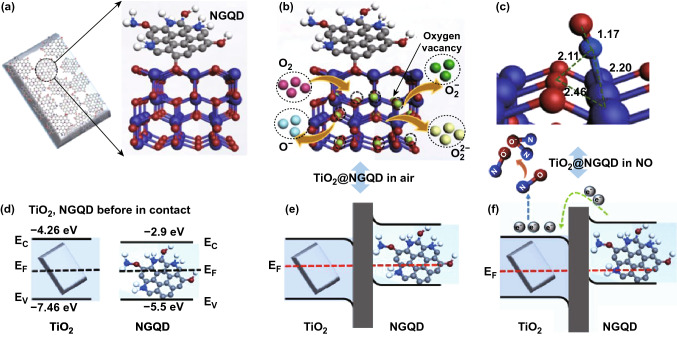
Schematic sensing mechanism of **a** TiO_2_@NGQDs hybrids, **b** O_2_ adsorption and conversion to oxygen ion species on TiO_2_ {001} surface, **c** NO adsorption on to TiO_2_ {001} surface. **d** Energy band structures of TiO_2_/NGQDs before in contact, and **e** formation of p–n junction after contact, and **f** electron transfer in the TiO_2_/NGQDs on exposure to NO. Reproduced with permission [76]. Copyright 2020, American Chemical Society

Very recently, Murali and co-workers demonstrated a UV-activated high-performance RT NO gas sensor based on nitrogen-doped graphene quantum dots (NGQDs) decorated TiO_2_ nanoplates with {001} facets exposed [76]. The response of the NGQDs/TiO_2_ hybrids without UV activation was improved from 12.0% to 100 ppm NO the decoration of NGQDs on TiO_2_, which dramatically enhanced the generation of electron–hole pairs due to good light absorption ability of NGQDs. The sensing mechanism is shown in Fig. 7. The bandgap alignment between NGQDs and TiO_2_ generates p–n junctions that can efficiently separate the electron–hole pairs. These p-n junctions promote the hot generated electron transfer from NGQDs to TiO_2_ and photogenerated holes transfer from TiO_2_ to NGQDs. In addition, the NGQDs also suggested promoting the formation of oxygen vacancies in the TiO_2_, which enhances the adsorption of oxygen ions and further facilitates their reaction with pre-adsorbed NO^−^. All these factors synergistically led to enhance the conversion efficiency of gas and carriers exchange, and charge separation, and which eventually improved sensing performance.

### IN_2_O_3_

In_2_O_3_ has been investigated for photoexcited gas sensors. Trocino and co-workers studied the effects of UV illumination on the recovery process of In_2_O_3_/PVP fibres after exposure to NO_2_ at RT [77]. They found that UV illumination could easily desorb the weakly bound adsorbed species, resulting in short recovery time. Nguyen et.al have reported the RT sensor performance of In_2_O_3_ nanostructure for detection of NO under UV illumination [78]. The sensor exhibited a sensitivity of 41.7 to 50 ppm NO and a response time of only 4 s because UV illumination promoted the NO (and O_2_) adsorption and desorption. Meanwhile, the response was observed to be affected by UV light intensity. Recently, Shen and co-workers demonstrated that mesoporous In_2_O_3_ nanorod arrays could detect NO_2_ at a ppb-level concentration at RT without UV illumination, but the sensor showed very poor recovery [79]. They showed that the recovery could be improved to 32 s by using UV illumination. In another work, Ma et al. achieved RT sensor performance from walnut-like In_2_O_3_ nanostructures to detect NO_2_ under UV illumination [80]. The sensor exhibits an ultrahigh sensitivity (219) towards 50 ppm NO_2_ with UV illumination. The studied showed that the high sensitivity of walnut-like In_2_O_3_ was mainly attributed to the effective participation of photogenerated electrons.

### WO_3_

In addition to ZnO and SnO_2_, WO_3_ has been also frequently studied for photoactivated gas sensors. According to Giberti, the increase in the conductivity WO_3_ gas sensor in the air was attributed to the photodesorption of surface oxygen under UV illumination [81]. RT sensing performance to detect NO_2_ enabled light illumination was by also reported. For example, Zhang et al. presented an RT NO_2_ gas sensor based on WO_3_ under visible light illumination [52]. It was found that the light wavelength and light intensity had a great influence on sensing characteristics. Under blue light (480 nm) illumination, the sensor exhibited a response of 2.9 to 160 ppb NO_2_ at RT, but the response/recovery time was long, i.e., 14.9/18.3 min. The improved sensing property was ascribed to the acceleration of the reactions by photo-energy under illumination.

Cantalini and co-workers studied the NO_2_ sensing performances of WO_3_ electrospun nanofibers both activated by thermal and light activation including red, green and blue light [82]. They showed that the baseline resistance in dry air was highly dependent on the lights, showing a decrease by switching from dark, red, green and blue light, respectively. Accordingly, the sensor response to 400 ppb NO_2_ was also improved from 9% (dark) to 38% (red), 55% (green) and 92% (blue). An interesting finding is that under thermal activation at 75 °C, the sensor response without light illumination is 18.4, which is higher than that (12.4) under blue light illumination, due to the light-activated desorption of adsorbed oxygen from WO_3_ surface.

**Fig. 8:**
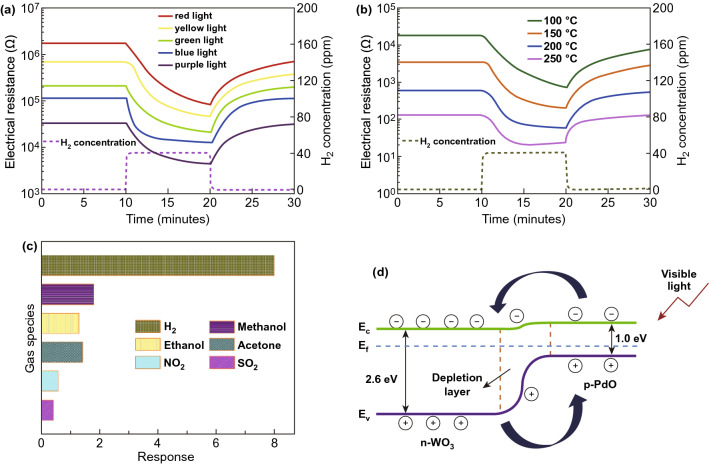
Resistance variation of the PdO-WO_3_ sensor to 40 ppm H_2_ at RT under **a** visible light illumination and **b** thermal heating at various temperatures. **c** Selectivity of the PdO-WO_3_ sensor towards 40 ppm H_2_, 300 ppm acetone, 300 ppm ethanol, 300 ppm methanol, 10 ppm NO_2_ and 100 ppm SO_2_ under blue light illumination at RT. **d** Schematic carrier excitation in the PdO-WO_3_ nanohybrids under visible light illumination. Reproduced with permission [83]. Copyright 2016, Elsevier

Apart from NO_2_, the photoactivated WO_3_ sensors have been used to detect H_2_. Zhang et al. reported a novel RT H_2_ sensor based on PdO loaded WO_3_ nanohybrids [83]. Their UV-Vis spectra revealed that PdO-WO_3_ sensor has a broader visible light absorption range compared with pureWO_3_. This resulted in the good responses to ppm-level H_2_ gas under visible light illumination (Fig. 8a), and the best performance was achieved with blue light, showing a response of 6.15 to 40 ppm H_2_ and the response/recovery time was 3.2/7.9 min. This performance is comparable to the result obtained under thermal activation at between 200 and 250 °C (Fig. 8b). It also has an excellent selectivity, as shown in Fig. 8c. The enhanced properties were attributed to the promotion effect of PdO, the heterojunction between PdO and WO_3_, as well as the photoactivation effect, as shown in Fig. 8d.

## Photoactivated Two-dimensional (2D) Materials

### Graphene

**Fig. 9:**
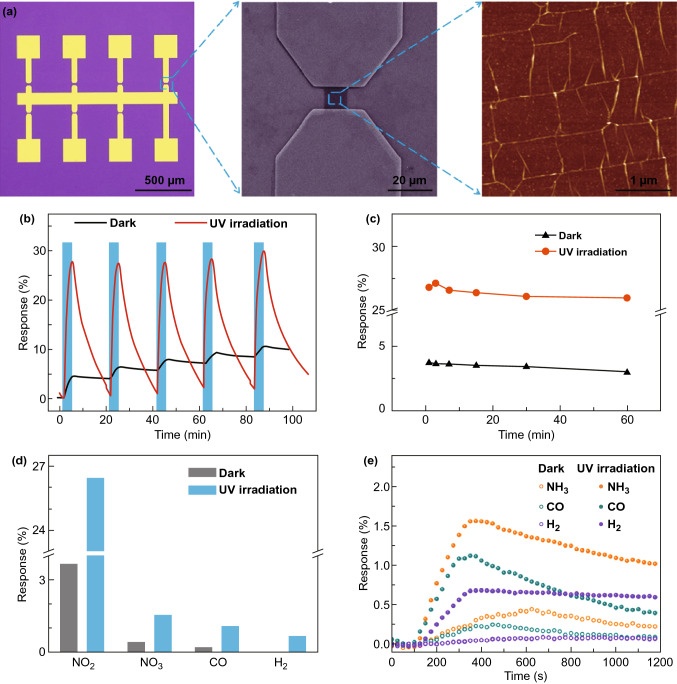
**a** Optical image of graphene sensors array, SEM image of graphene device, and AFM image of graphene. **b** Response and **c** stability test of graphene sensor to 100 ppm NO_2_ in dark and UV illumination. **d** Selectivity bar diagram, and **e** transient response of graphene sensor to NH_3_, CO and H_2_ at room temperature in dark and UV illumination. Reproduced with permission [87]. Copyright 2019, American Chemical Society

Since the isolation of graphene in 2004 by Novoselov and Geim, graphene has become a promising candidate for advanced electronic device applications owing to its excellent electrical, mechanical and chemical properties [84–86]. The same group fabricated the first graphene gas sensor in 2007 and observed that graphene can detect gases at RT and has the potential to detect even a single gas molecule [28]. Despite the excellent gas adsorption, the fast response and complete desorption of the gas molecules from the surface of graphene at RT are main issues which have been addressed from light illumination or heating the device using micro-heater. Ma and co-workers studied the effects of thermal and optical energy on gas sensing characteristics of graphene via fabricating graphene sensor array (Fig. 9a) [87]. They observed that transferred CVD grown graphene-based gas sensor exhibited deterioration in sensitivity to NO_2_ gas with increased temperature (25–100 °C). The high temperature increased the desorption rate of NO_2_ molecules, which results in less adsorption of gas molecules [88]. However, UV light irradiation enhanced the sensitivity of the sensor sevenfold and completed the incomplete recovery with decreased recovery time about fivefold compared to that of in dark condition at RT (Fig. 9b, c). Moreover, the sensor showed reliable selectivity to NO_2_ gas against many other gases via photoactivation as shown in (Fig. 9d, e). The sensitivity was enhanced due to excess photogenerated electrons and availability of a large number of adsorption sites for more number of NO_2_ molecules thorough cleaning of graphene surface from pre-adsorbed ambient oxygen ions or water molecules. Moreover, complete recovery at RT was achieved by accelerating desorption rate of NO_2_ molecules via light energy. Likewise, Harutyunyan et al. enhanced the sensitivity of the graphene gas sensor via in situ cleaning of graphene by UV light [89]. The pristine graphene sensor exhibited unprecedented sensitivity with a detection limit of 158 ppq, 2.06 ppt and 33.2 ppt to NO, NO_2_ and NH_3_ gases at RT. This ultra-sensitivity at RT was attributed to the cleaning of graphene via continuous in situ UV light illumination under inert atmosphere (under N_2_ gas ambient).

Further, Lai et al. enhanced acetone sensing properties of graphene sensor via UV light irradiation with optimized spacing between electrodes of resistive sensor device [90]. In this work, they fabricated different resistive gas sensor devices having electrodes spacing of 50, 100, 200, and 400 µm by using transferring CVD grown graphene on a glass substrate. The sensor with 400 µm electrodes spacing exhibited two times higher sensitivity to acetone in a range of 100 to 1000 ppb than that of the 50 µm spacing electrodes sensor device. This improved sensitivity by large electrodes spacing was attributed to a combination effect of tensile strain on graphene, doping effect of glass and increased surface area with many defects at grain boundaries. Moreover, the sensitivity was enhanced to acetone by seven times under UV illumination at RT with response/recovery time of 300 s through desorption of natural atmospheric oxygen and water molecules from the surface of the graphene. The same group also improved the response/recovery kinetics of the graphene sensor to NO_2_ gas at RT by using rapid thermal annealing (RTA) and UV light irradiation [91]. The as-fabricated sensor device using transferred CVD grown graphene was treated via RTA at 300 °C in N_2_ environment for providing more adsorption surface area through removing the polymer residue of transfer process. This sensor exhibited four times more sensitivity to NO_2_ than that of pristine graphene sensor without RTA treated. However, the sensor’s incomplete recovery at RT was improved to complete by UV illumination during recovery time.

To further improve the gas sensing performance of the photoactivated graphene sensors, graphene sensing layer was decorated with noble metals, metal oxide and polymer nanoparticles. Chu et al. fabricated a photoactivated NH_3_ RT gas sensor by depositing different thickness of Ti on the graphene surface [92]. The optimized 5 nm thickness of Ti on the graphene surface was oxidized in terms of titanium oxide with different Ti valances and lower valances helped to reduce optical bandgap. Visible light was sufficient to create electron–hole pairs and these photoexcited electron–hole pairs as well as synergistic catalysis effects of TiOx/graphene assisted to improve the sensitivity of the sensor to NH_3_ gas with complete recovery (2.5 min) at RT under visible light irradiation. Further, Wu et al. enhanced the sensitivity of graphene sensor to NO_2_ gas at RT under visible light irradiation via decorating polymer (polystyrene (PS)) beads on graphene surface [93].

**Fig. 10:**
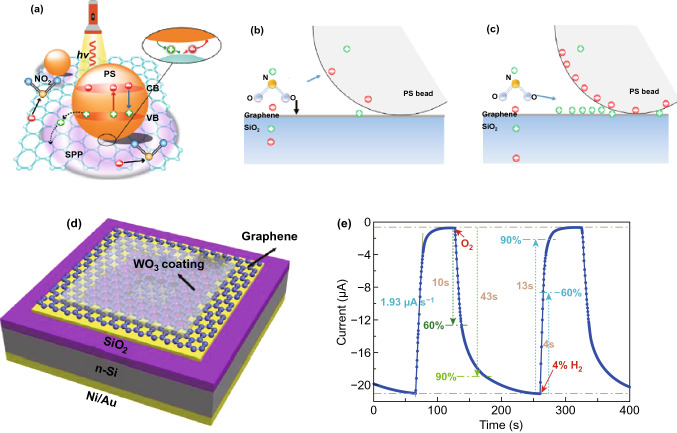
**a** Schematic representation of charge transfer in between graphene and PS bead under light illumination. The electric potential and static forces on adsorbed NO_2_ molecules on graphene/PS hybrid under **b** dark condition and **c** light illumination. Reproduced with permission [93]. Copyright 2019, American Chemical Society. **d** Schematic illustration of gasochromic-Pd-WO_3_/graphene/Si tandem structure of hydrogen sensor. **e** The transient gas response of the sensor to H_2_ gas under light illumination. Reproduced with permission [102]. Copyright 2018, Elsevier

There was electron transfer from graphene to PS beads at the graphene/PS interface and some exciting surface plasmon polaritons are also present in graphene through diffraction of light on microbeads (Fig. 10a), which helped to enhance the sensitivity of graphene sensor with a detection limit of 0.5 ppb NO_2_ at RT under laser illumination. Figure 10b, c clearly illustrates the concave region at PS bead/graphene interface and upon NO_2_ exposure, two static forces, one from the bead and another from graphene drag more number of gas molecules which results in enhanced gas response in PS decorated graphene than that of the pristine graphene sensor. Moreover, under light illumination, photoexcited electrons transferred from graphene to PS bead and thereby, a dipole layer was formed at PS/graphene interface. As a result, dipolar interaction was occurred in between the dipole layer and polar NO_2_ molecules. This dipolar interaction under light illumination was stronger from static interaction in dark condition and which was helpful to enhance sensitivity and fast adsorption to NO_2_ gas at RT. Likewise, Banihashemian et al. reported enhanced ammonia detection by using Ag particles decorated graphene sensor at RT under blue LED (10 mW cm^−2^) exposure [94]. The enhancement in sensitivity was attributed to surface plasmon resonance and spillover effects. Besides the decoration of graphene surface via nanoparticles, different nanocomposites and hybrids of graphene such as WO_3_ nanorodes/Graphene [95], carbon nitride/rGO [96], RGO-CeO_2_ [97], WO_3_/rGO [98], MoS_2_/rGO [99], p-phenylenediamine-graphene oxide (PGO)/InGaN [100], Pd-decorated ZnO/rGO [101] and Pd-WO_3_/graphene/Si [102] were utilized for improving the gas sensing performance of graphene sensor at RT under light irradiation. Zhang et al. designed a gasochromic-Pd-WO_3_/graphene/Si tandem structure (Fig. 10d) for hydrogen sensing at RT under light irradiation [102]. In this structure, Pd-WO_3_ and graphene/Si worked as sensing and photodetector layer through utilizing their gasochromic and photovoltaic properties, respectively. Upon hydrogen exposure, Pd dissociated the H_2_ into H atoms and WO_3_ converted to H_x_WO_3_ which decreased the transmittance and was synchronously sensed by graphene/Si photodetector and that changed its photocurrent corresponding to H_2_ concentration. Thus, the sensor detected even low concentration of 0.05 vol% H_2_ with fast response time (13 s) and recovery time (43 s) at RT under laser (980 nm) illumination as shown in Fig. 10e.

**Fig. 11:**
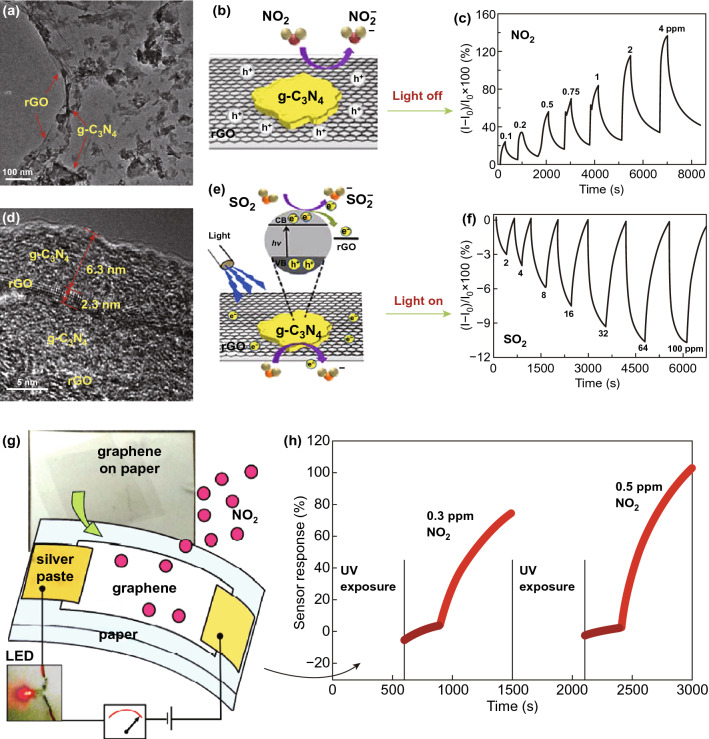
SEM image of g-C_3_N_4_/rGO hybrid **a** in low, and **d** high magnification. **b** Schematic representation of charge transfer between g-C_3_N_4_/rGO hybrid and NO_2_ under dark condition. **c** Transient gas response of the hybrid sensor to NO_2_ gas under dark condition. **e** Schematic representation of charge transfer between g-C_3_N_4_/rGO hybrid and SO_2_ under UV light illumination. **f** Transient gas response of the hybrid sensor to SO_2_ gas under UV light illumination. Reproduced with permission [103]. Copyright 2017, American Chemical Society. **g** Schematic illustration of a graphene sensor on paper substrate. **h** Gas response of the flexible graphene sensor. Reproduced with permission [104]. Copyright 2015, American Chemical Society

Besides the high sensitivity and fast response/recovery kinetics, selectivity of the sensor is also one of the most important aspects for the usage of the sensor on the commercial sensing platform. In this context, Wu et al. reported a ‘light on and off’ strategy for selective detection of NO_2_ and SO_2_ gas by using the 2D g-C_3_N_4_/rGO van der Waals heterostructure [103]. In this work, a layer-by-layer self-assembly approach was used for fabricating g-C_3_N_4_/rGO stacking hybrid on a paper substrate (Fig. 11a, b, d, e). The p-type semiconducting g-C_3_N_4_/rGO sensor under light off condition exhibited no response to SO_2_ and high sensitivity to NO_2_ gas with detection as low as 100 ppb at RT (Fig. 11c). In contrast, under UV light irradiation, the sensor with changed n-type semiconducting behaviour showed sensitivity to SO_2_ with detection as low as 2 ppm, as shown in Fig. 11f. Under UV irradiation, photon energy excited the electrons in valance band of g-C_3_N_4_, and then, these photoexcited electrons transferred into rGO and a negative charge layer formed on the surface. Thereby, SO_2_ gas molecules extracted electrons which results in negative response through decreasing electrons concentration in the g-C_3_N_4_/rGO. This approach to distinguish the NO_2_ and SO_2_ gas via light source was attributed to effective charge transfer between g-C_3_N_4_ and rGO. Likewise, Ren et al. also reported UV light-activated gas sensor for selective detection of NO_2_ and SO_2_ gas by using a nanocomposite of SnO_2_ nanofibers and rGO [74]. On the other hand, high flexibility and transparency aspects of graphene make it a leading candidate for emerging flexible and wearable gas sensing technology.

In this regard, Raghavan et al. [104] demonstrated deep UV light-activated flexible graphene sensor for NO_2_ detection at RT. They directly transferred the CVD grown graphene on paper without any intermediate layers and called it G-paper (Fig. 11g) which showed a detection limit of 300 ppt to NO_2_ at RT. Under deep UV light irradiation, fast response and recovery time were achieved at RT due to cleaning of graphene through desorption of atmospheric adsorbents (Fig. 11h) and this also was confirmed by Raman spectroscopy with indicating reduction of p-type doping in G-paper. Besides the high gas sensing performance of the sensor, this method is very useful for biodegradable and wearable sensor applications due to simplicity, low cost and high productivity.

**Fig. 12:**
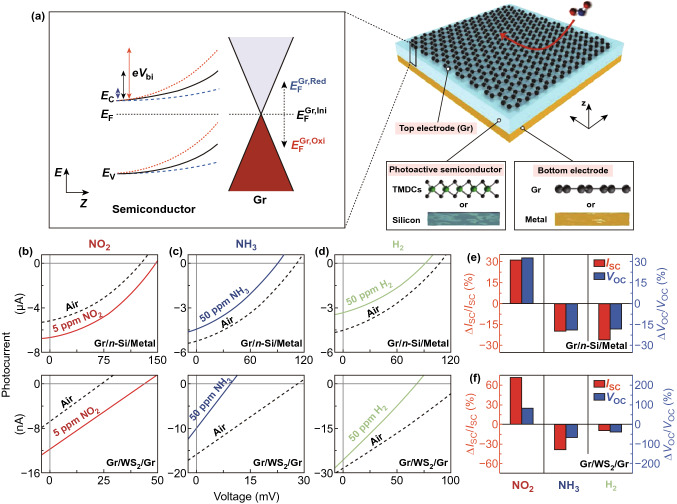
**a** Schematic representation of a self-powered chemical sensor fabricated by graphene-based photovoltaic heterojunction device (right) and energy band diagram of the device (left). Change in photocurrent of graphene/Si/metal and graphene/WS_2_/graphene device under **b** NO_2_, **c** NH_3_ and **d** H_2_ ambient. Histograms of the relative percentage change in photocurrent (red) and photovoltage (blue) to NO_2_, NH_3_ and H_2_ gases of **e** graphene/Si/metal and **f** graphene/WS_2_/graphene device under white light illumination. Reproduced with permission [105]. Copyright 2018, WILEY‐VCH Verlag GmbH & Co. KGaA, Weinheim

It is noted from all above results that photoactivation removes the heating element from the graphene sensors to achieve fast response and complete recovery at RT and it also improves the sensitivity and selectivity of the graphene sensors. However, chemiresistive sensor required external supply voltage or current to electrical readout and so, it consumed electrical power for its operation. Nowadays, chemical sensors consuming ultralow-power are needed for its usage in the Internet of things applications. Lee et al. reported a self-powered chemical sensor fabricated by a graphene-based heterojunction device [105]. In this work, photovoltaic heterojunctions were fabricated via contact of top graphene layer with photoactive materials silicon (Si) or tungsten disulphide (WS_2_) as shown in Fig. 12a. Upon gas exposure, the electrochemical potential of graphene was changed owing to the one-atom-thick layer, which results in modulation of built-in potential at the interface of graphene and Si or WS_2_. Thereby, change in photocurrent or photovoltage of the device was measured at RT without applying external bias. As a result, the sensor showed good response to NO_2_, NH_3_ and H_2_ gases with detection as low as 1 ppm H_2_ at RT (Fig. 12b–f).

### MoS_2_

**Fig. 13:**
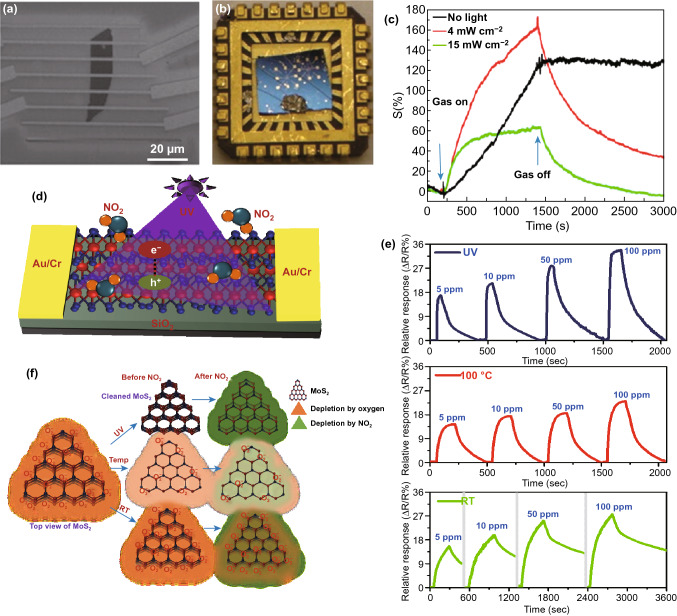
**a** SEM image of mechanically exfoliated MoS_2_-based device. **b** MoS_2_ device mounted on the chip. **c** Sensitivity to NO_2_ gas of the MoS_2_ sensor under different intensities of the green light source. Reproduced with permission [112]. Copyright 2013, American Chemical Society. **d** Schematic representation of a CVD grown MoS_2_ sensor under UV light illumination. **e** Transient gas response to different concentrations of NO_2_, and **f** gas sensing mechanism, of the CVD grown MoS_2_ sensor at room temperature, 100 °C and under UV illumination. Reproduced with permission [114]. Copyright 2017, American Chemical Society

MoS_2_ is one of the most famous members of layered transitional metal dichalcogenides (TMDCs) family for electronics device applications [106–108] and particularly, gas sensor devices owing to its unique electrical and physical properties [24, 34, 109–111]. MoS_2_ sensor has attracted immense attention in gas sensing field under light illumination due to its excellent optoelectronics aspects. Late et al. performed a gas sensing experiment on mechanically exfoliated five layers MoS_2_ based gas sensor under the green light illumination (532 nm) [112]. The SEM image of the MoS_2_ device and mounted device on a chip are shown in Fig. 13a, b. They measured the sensitivity to each 100 ppm NO_2_ and NH_3_ upon exposure to green light with different optical powers. The sensitivity of the sensor was enhanced up to optimal irradiation power and suddenly decreased for high irradiation power, as shown in Fig. 13c. This behaviour was similar to photoactivated metal oxide-based gas sensor. Likewise, Friedman et al. also measured the sensitivity of mechanically exfoliated MoS_2_ sensor under the illumination of white light at RT [113]. They observed that the sensor exhibited about 10 times higher sensitivity to trimethylamine upon exposure to light than that of switched off light condition. However, gas sensing mechanism of enhancing the sensitivity of the MoS_2_ gas sensor under the illumination of the light source was not clear. Therefore, many research efforts have been attempted to elucidate the improvement in gas sensing characteristics of photoactivated MoS_2_. Kumar et al. demonstrated gas sensing performance of CVD grown multilayer MoS_2_ at RT under UV illumination [114]. The sensor showed high selectivity towards NO_2_ against many other gases (CO_2_, NH_3_, CH_4_, H_2_ and H_2_S) under UV illumination. Optical energy assisted to provide more numbers of adsorption active sites on the surface of MoS_2_ through desorption of ambient oxygen and contamination because photogenerated holes reacted with pre-adsorbed oxygen ions and formed O_2_ gas as shown in Fig. 13f. On the other hands, thermal energy decreased the sensitivity of the sensor to NO_2_ gas because thermoactivation accelerated the desorption rate than adsorption rate (Fig. 13e) [115, 116]. In addition, CVD grown monolayer MoS_2_ also showed enhanced sensitivity to NO gas at RT under UV light (254 nm) [117].

From the above all reports, it is clear that light source was switched on during throughout gas sensing experiment and this optical energy enhanced the sensitivity of the MoS_2_ gas sensor to different gases and gas sensing mechanism under the light illumination also was proposed. However, complete recovery at RT under light illumination was still vague. In this context, Kim et al. proposed a complete recovery mechanism for MoS_2_ gas sensor via illumination of light during recovery process [118]. Under UV illumination, photogenerated hole reacted with adsorbed NO^-^, which results in NO_2_ desorption through changing its chemical state. Simultaneously, photogenerated electrons decrease the resistance value of the MoS_2_ sensor, and thereby, the sensor achieved its initial baseline resistance value. Thus, photogenerated electron–hole pairs helped to obtain complete recovery at RT without raising the temperature of the MoS_2_ sensor. Moreover, this proposed mechanism also verified by Raman and PL experiments.

However, gas sensing mechanism of MoS_2_ gas sensor under light illumination is needed to further explain quantitatively in the context of a number of adsorption sites and adsorption energy values.

**Fig. 14:**
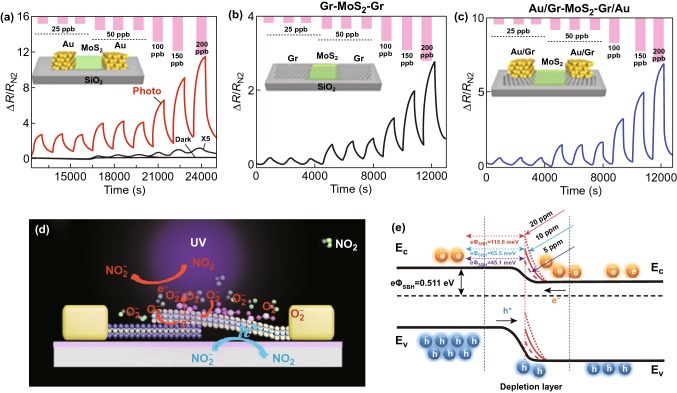
Gas response to different concentrations of NO_2_ gas at room temperature under red light illumination of **a** Au/MoS_2_/Au, **b** Gr/MoS_2_/Gr, and **c** Au/Gr-MoS_2_-Gr/Au device. Reproduced with permission [120]. Copyright 2019, American Chemical Society. **d** Schematic representation of an n–p-type van der Waals homojunction of MoS_2_ under UV illumination. **e** Gas sensing mechanism of the n–p-type van der Waals homojunction of MoS_2_ via energy band diagram. Reproduced with permission [126]. Copyright 2020, WILEY‐VCH Verlag GmbH & Co. KGaA, Weinheim

In order to improve gas sensing performance of MoS_2_ sensor under light illumination, some suitable approaches and strategies were adopted by exploiting structure and interface engineering. Chueh et al. reported the detection of NO gas at ppb level using 3D cone-shaped MoS_2_ bilayers under indoor light illumination [119]. In this work, 3D cone-shaped MoS_2_ bilayers were fabricated by sulphurizing 2-nm-thick MoO_3_ film on pre-patterned 2” cone patterned sapphire substrate. The sensor exhibited sensitivity of ~189.2%/ppm with detection as low as ~0.06 ppm NO at RT under UV light illumination. Moreover, this 3D structure of MoS_2_ showed about twofold higher sensitivity than that of flat MoS_2_ sensor. The enhancement in sensitivity of 3D architecture of MoS_2_ under light source was attributed to 30% increased surface area as well as enhanced light absorption through light scattering effects. In addition, electrode’s materials also play a crucial role in tuning the sensing performance of chemiresistive/FET- type gas sensor. From this view, Mulchandani et al. reported ultrasensitive optoelectronic NO_2_ gas sensor using special arrangements in the electrode’s materials of FET-type MoS_2_ sensor (Fig. 14a–c) [120]. The Au electrodes-based Au/MoS_2_/Au sensor exhibited excellent sensitivity 4.9%/ppb (4900%/ppm) at RT under red light illumination. In contrast to Au/MoS_2_/Au sensor, Au coated graphene (Gr) electrode-based Au/Gr-MoS_2_-Gr/Au sensor showed ultra-sensitivity to NO_2_ with detection as low as 0.1 ppb concentration at RT.

Incorporation of another material into MoS_2_ in terms of nanocomposite or hybrid as a new sensing material also used for enhancing the gas sensing performance through chemical and electronic sensitization effects [14, 121]. Controlled Au nanoparticles functionalization changed carrier concentration of MoS_2_ through electrons transferring from Au to MoS_2_ [109]. This controlled n-type doping effect helped to discriminate hydrocarbon- and oxygen-functional group based VOCs by showing different sensing behaviour. Pristine MoS_2_ exhibited increase resistance value upon exposure to all VOCs, but Au:MoS_2_ showed decrease resistance value to oxygen-functionalized compounds and the same increase resistance value behaviour to hydrocarbon-based VOCs. Likewise, Jung et al. demonstrated different sensing behaviour to oxygen-functionalized VOCs via functionalization of the MoS_2_ by a thiolated ligand (mercaptoundecanoic acid (MUA)) [122]. The MUA-conjugated MoS_2_ showed a negative response to oxygen-functionalized VOCs but, pristine MoS_2_ exhibited a positive response to the same VOCs at RT. Further, Guo et al. improved the gas sensing characteristics of decorating Au nanoparticles on the surface of MoS_2_ under UV light illumination [123]. The Au-MoS_2_ gas sensor exhibited about three times higher response to NO_2_ with complete recovery at RT under UV illumination than that of in the dark condition. The enhancement in sensitivity was attributed to an increased number of active adsorption sites as well as introducing active catalysts via Au nanoparticles. Moreover, Au nanoparticles accelerated trapping of more numbers of photons which generated additional photoexcited charge carriers for more gas–solid interaction. Under UV illumination, effective separation of photoexcited charge carriers at MoS_2_/Au interface due to different work function of MoS_2_ and Au was also helpful to contribute for obtaining fast full recovery at RT. Guo et al. reported an ultrasensitive UV-assisted NO_2_ gas sensor based on a nanocomposite sensing layer of MoS_2_ and ZnO nanowires [124]. The MoS_2_/ZnO sensor showed excellent sensitivity of 0.93/ppb with a detection limit of 50 ppq and complete recovery at RT under UV illumination. This improved performance of the sensor under optical energy was the results of two reasons. On the one hand, under UV illumination, additional photogenerated charge carriers react with more number of NO_2_ molecules. On the other hand, a large number of MoS_2_/ZnO nanoheterojunctions helped for extension of depletion region and photoexcited electrons moved into ZnO from the conduction band of MoS_2_, while excited hole transferred into MoS_2_ from valance band of ZnO. This effective separation of charge carriers improved the sensing characteristics by avoiding charge recombination at the interface. To further enhance the sensing characteristics, Wang et al. fabricated a near-infrared (NIR) optoelectronic NO_2_ gas sensor using a nanocomposite of ZnO quantum dots decorated sulphur vacancy-enrich MoS_2_ (Sv-MoS_2_) [125]. Sulphur vacancy introduced new energy levels between conduction and valance band of MoS_2_. These localized levels helped to increase photoexcited charge carriers and charge transfer by absorbing more light photons under NIR illumination. As a result, the Sv-MoS_2_/ZnO sensor exhibited high sensitivity of 226% to 200 ppb NO_2_ at RT under NIR illumination. Moreover, the sensor also showed fast response and recovery time (75 and 111 s) at RT.

The optoelectronic gas sensors based on van der Waals heterostructures are recently attracting enormous attention for developing high-performance gas sensor. Van der Waals heterostructures owing to its strong light matter interaction and tuning of carrier concentration or energy band diagram by electrical, magnetic and optical energy render them a promising candidate for optoelectronic gas sensors. Despite the huge potential of heterostructures of MoS_2_ in gas sensing field, there are still few reports of MoS_2_ heterostructures-based optoelectronics gas sensors and it is in the nascent stage. Recently, Zhang and co-worker reported a highly selective NO_2_ gas sensor using 2D planar van der Waals p-n homojunction of MoS_2_ under UV illumination [126]. In this work, n-type and p-type MoS_2_ were fabricated by CVD and sol-gel process, respectively (Fig. 14d). The p-n van der Waals homojunction of MoS_2_ exhibited about 60 times higher sensitivity to 20 ppm NO_2_ than that of the individual p-type MoS_2_. Moreover, the sensor showed a low detection limit of 8 ppb with very fast complete recovery (< 30 s) at RT under the UV illumination. This excellent sensing performance of van der Waals-based sensor was attributed to modulation of barrier height at the p-n junctions of MoS_2_ upon exposure to NO_2_ gas (Fig. 14d, e). Further, Kim et al. demonstrated NO_2_ gas sensor under UV light illumination using 2D/3D heterostructure of n-MoS_2_/p-GaN [127]. The sensor showed high sensitivity of 98.42% to 50 ppm NO_2_ with complete recovery at RT under UV illumination with applied reverse bias. Besides the well-known mechanism of heterostructures as modulation of barrier height at heterojunctions upon exposure to gas molecules, reverse bias strategy also utilized here to enhance the sensing performance of heterostructure sensor through improving the photoextraction from the p-n junction [128].

**Fig. 15:**
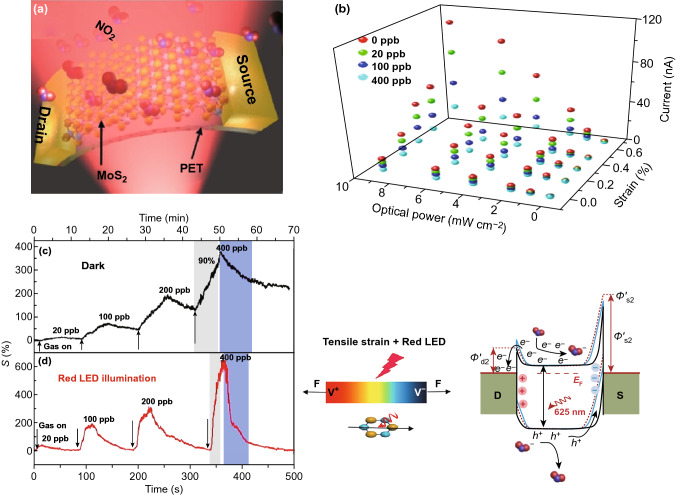
**a** Schematic representation of a flexible MoS_2_ sensor under red light illumination. **b** Device current in different concentrations of NO_2_ gas ambient under different optical power of red light illumination with an applied different strain. Gas response to different concentrations of NO_2_ gas of the flexible sensor under **c** dark and **d** red illumination. **e** Gas sensing mechanism of the flexible MoS_2_ sensor via energy band diagram under tensile strain and red illumination. Reproduced with permission [129]. Copyright 2018, Science China Press. Published by Elsevier B.V. and Science China Press

All the above reports are limited to optoelectronic MoS_2_ gas sensors on rigid substrates; nonetheless, excellent flexibility and mechanical properties of the MoS_2_ render it a promising candidate for flexible and wearable sensors applications. Wang et al. reported a high-performance flexible MoS_2_ gas sensor at RT by exploiting photogating and piezo-phototronic effects [129]. Figure 15a, b illustrates the schematic of the flexible device and 3D representation of the current response of the sensor to NO_2_ gas under different optical powers and tensile strain. The sensor exhibited excellent sensitivity of 671% to 400 ppb NO_2_ at RT under red light (625 nm) illumination with 0.67% tensile strain (Fig. 15d). Moreover, the sensor showed dramatically improved response time of 16 s and complete recovery time of 65 s at RT. The excellent sensing performance of the sensor was attributed to tuning the Schottky barrier height at two back-to-back Pd-MoS_2_ junctions upon exposure to gas molecules via a combination of photo-gating and piezo-phototronic effects (Fig. 15e).

### MOTe_2_

Molybdenum ditelluride (MoTe_2_) is an emerging material in TMDCs family and has a lower energy bandgap of ~ 1.0 eV than other semiconducting TMDCs materials. Due to a smaller bandgap, MoTe_2_ showed photodetection in a wider range from visible to near-infrared wavelengths [130, 131]. Besides the excellent optoelectrical properties, larger bond length and lower binding energy of MoTe_2_ are important aspects for utilizing it in the optoelectronic gas sensing field. Zhang et al. reported enhancement in sensitivity of MoTe_2_ gas sensor via continuous illumination of light throughout the gas sensing experiment [132].

**Fig. 16:**
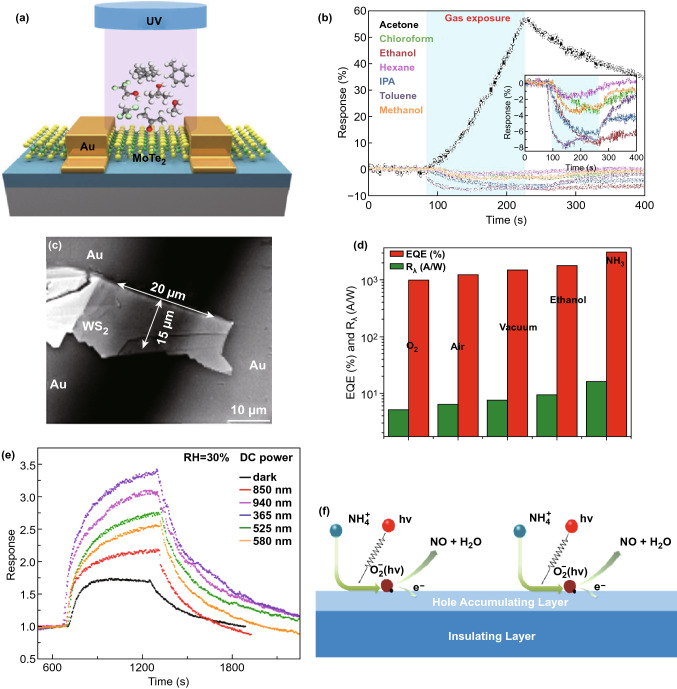
**a** Schematic representation of a MoTe_2_ sensor under UV light illumination. **b** Gas response to different VOCs under UV illumination. Reproduced with permission [40]. Copyright 2018, American Chemical Society. **c** SEM image of mechanically exfoliated WS_2_ flake. **d** Photoresponsivity and external quantum efficiency of the WS_2_ sensor under light illumination in different gas ambient. Reproduced with permission [135]. Published by Nature Publishing Group. **e** Gas response of the sensor to NO_2_ gas under different wavelengths of light sources. **f** Gas sensing mechanism of the sensor under light illumination. Reproduced with permission [136]. Copyright 2017, Elsevier

In this work, the MoTe_2_ sensor was fabricated by mechanical exfoliation, and interestingly, MoTe_2_ device converted its p-type semiconducting behaviour into n-type after continuous illumination of UV light for 2 h in an N_2_ environment. This changed behaviour under UV illumination was attributed to the removal of contamination of impurity molecules (O_2_ and H_2_O). The n-type MoTe_2_ sensor showed increase sensitivity to NH_3_ gas under illumination with reducing wavelength sources (near-infrared-red-to-UV region). Further, the sensor showed a rapid increase in sensitivity to NH_3_ gas with increased intensity from 0.25 to 1 mW/cm^2^ of UV light source (254 nm) and saturation trend in sensitivity for increased intensity up to 2.5 mW cm^−2^. As a result, the sensor exhibited excellent sensitivity about 25 times more with a low detection limit of 3 ppb NH_3_ gas under UV illumination with an intensity of 2.5 mW cm^−2^. Also, the same group used the as-fabricated p-type MoTe_2_ gas sensor for NO_2_ detection under UV light illumination [133]. The sensor dramatically exhibited enhanced sensitivity of 58-1744% to 20-300 ppb NO_2_ with an extraordinary low detection limit of 123 ppt under UV illumination (254 nm). Moreover, the sensor showed complete recovery within 5 min at RT through accelerating desorption rate of NO_2_ via photoactivation of MoTe_2_. They suggested three reasons for enhancing the sensitivity of the sensor. First, p-type behaviour of MoTe_2_ is more sensitive to oxidizing gas (NO_2_) which extracts a large number of electrons from MoTe_2_ and which results in shifting of Fermi level towards valence band. Thereby, holes easily tunnel from decreased Schottky barrier. Second, photon energy desorbed pre-adsorbed ambient oxygen ions from the surface of MoTe_2_, and therefore, a large number of availability of adsorption sites enhanced the adsorption of more number of NO_2_ molecules. Third, photoexcited plasmons promote molecular desorption because UV light wavelength of 254 nm lies in strong optical absorption window of MoTe_2_ owing to π-electrons plasmon excitation. Moreover, photogenerated holes react with adsorbed NO_2_^-^ and formed NO_2_ gas during recovery process. Further, the same group also used p-type MoTe_2_ FET-type gas sensor for discriminating ketone compounds with high sensitivity from other volatile organic compounds (VOCs) by the influence of UV light (Fig. 16a) [40]. The sensor exhibited excellent sensitivity to acetone with a low detection limit of 0.2 ppm at RT under UV illumination. The sensor showed a negative response to all VOCs in dark condition because electrons donor behaviour of VOCs decreased hole carriers concentration in p-type MoTe_2_. Surprisingly, under UV illumination, the sensor showed positive response to acetone and same negative response to all other VOCs (Fig. 16b). This type of opposite response to acetone was also observed in MoS_2_ and ReS_2_ sensors with the influence of UV light. An acetyl group in ketone compound enhanced UV absorption of 254 nm wavelength which stimulated strong photon–electron interaction within molecules, resulting in change behaviour of acetone from reducing to oxidizing. That change was responsible to show positive response to acetone under UV illumination, while the sensor exhibited negative response in dark condition.

### WS_2_

Excellent photoelectrical properties of tungsten disulphide (WS_2_) [134], a member of TMDCs family, make it a promising candidate for optoelectronic gas sensor. Li et al. fabricated a field-effect transistor using mechanically exfoliated multilayer WS_2_ (Fig. 16c) and measured the photoelectrical properties under the influence of different gases molecules [135]. Under the illumination of red light (633 nm) approximate to WS_2_ bandgap, the device showed a change in its responsivity (*R*_λ_) and external quantum efficiency (EQE) for both oxidizing and reducing gases at RT. This change was attributed to perturbation in charge carrier density in WS_2_ by charge transfer between WS_2_ and physical-adsorbed gas molecules. The oxidizing gas (O_2_) as ‘p-dopants’ extracted photogenerated electrons from WS_2_ and reduced the (*R*_λ_) and EQE of the device. In contrast, reducing gas (ethanol, NH_3_) as ‘n-dopants’ contributed electrons into photoactivated WS_2_ and enhanced the (*R*_λ_) and EQE value as shown in Fig. 16d. As a result, the device exhibited maximum (*R*_λ_) and EQE value of 884 A W^−1^ and 1.7 × 10^5^%, respectively, under NH_3_ ambient due to strong electronic interaction between NH_3_ and WS_2_. Further, Gaskov et al. demonstrated sensing performance of WS_2_ gas sensor under a wide wavelength range from UV to near-infrared light (Fig. 16e) [136]. Among these light sources, the sensor exhibited the highest response of 3.4 to 10 ppm NH_3_ with fast response and recovery time at RT under UV light illumination (365 nm). The enhancement in the response under UV light was attributed to the orbital mixing theory. Due to optical energy, electrons in the highest occupied molecular orbital (HOMO) of NH_3_ on the N atom were excited and transferred to WS_2_ (Fig. 16f). Thereby, the response was increased under UV illumination compared to that of dark condition.

In addition to enhancing the sensitivity of WS_2_ under light illumination, some research efforts such as noble metal decoration and incorporation of another material into WS_2_ in terms of nanocomposites or hybrid are also used. Goodilin et al. decorated plasmonic Au nanoparticles on WS_2_ nanotubes (NT-WS_2_) and examined the sensing behaviour under the illumination of 530 nm LED source [137]. The Au-NT-WS_2_ sensor exhibited higher sensitivity in a range of 0.25–2.0 ppm NO_2_ at RT than that of pristine NT-WS_2_. Gas sensing mechanism was attributed to physisorption-charge transfer between NO_2_ and NT-WS_2_. Further, Cantalini et al. reported a high-performance NO_2_ gas sensor using WS_2_-rGO hybrids under purple-blue light (430 nm) illumination [138]. The sensor showed excellent sensitivity to NO_2_ with a low detection limit of 400 ppb and fast response and recovery kinetics.

### SnS_2_

**Fig. 17:**
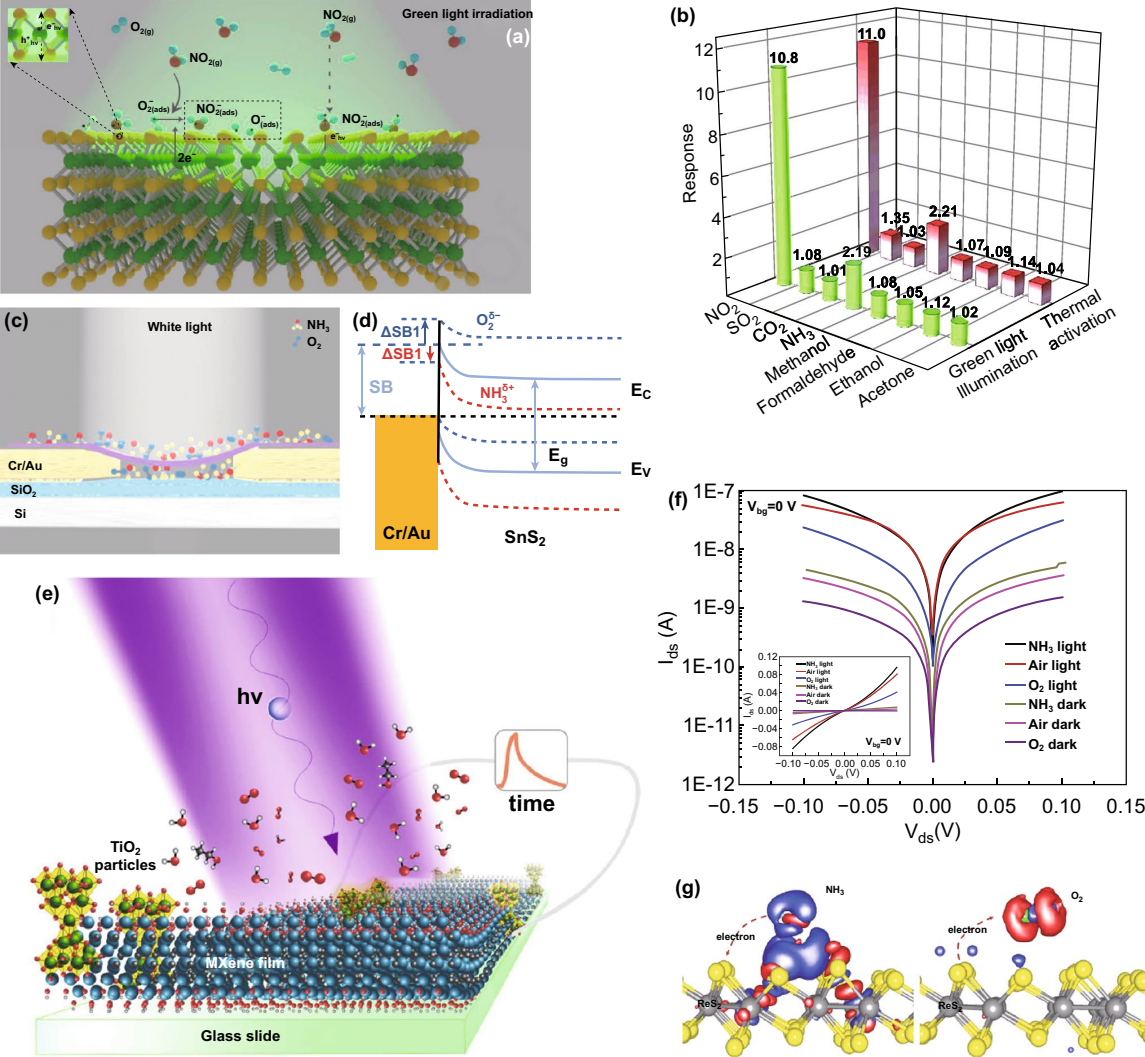
**a** Schematic representation of gas sensing mechanism of SnS_2_ sensor under green light illumination. **b** Gas response to different gases of the sensor under green light illumination and thermal activation. Reproduced with permission [143]. Copyright 2019, Elsevier. **c** Schematic illustration of the suspended SnS_2_-based device under white light illumination. **d** Gas sensing mechanism of the SnS_2_ sensor based on modulation of Schottky barrier upon exposure to NH_3_. Reproduced with permission [145]. Copyright 2018, WILEY‐VCH Verlag GmbH & Co. KGaA, Weinheim. **e** Schematic of MXene gas sensor under UV light illumination. Reproduced with permission [152]. Copyright 2018, American Chemical Society. **f** Current versus voltage of the suspended ReS_2_ sensor in different gas ambient. **g** Charge transfer between ReS_2_ and NH_3_ or O_2_. Reproduced with permission [148]. Copyright 2016, WILEY‐VCH Verlag GmbH & Co. KGaA, Weinheim

Tin disulphide (SnS_2_) is an n-type semiconducting layered material, and its structure is similar to members of TMDCs family [139–142]. Naturally abundant, higher electronegativity than TMDCs materials and wider direct bandgap (2.1 eV) have been attracted attention for the usage of SnS_2_ in optoelectronic gas sensing applications. Gu et al. reported a RT NO_2_ gas sensor of SnS_2_ under green light illumination [143]. A chemiresistive sensor was fabricated using SnS_2_ nanosheets which were synthesized via a simple one-step hydrothermal method. Under green light, the sensor exhibited reliable selectivity towards NO_2_ with detection as low as 38 ppb concentration and also showed a fast response and complete recovery at RT. Improvement in the sensing performance of the sensor was ascribed to increased carriers concentration in SnS_2_ via photon energy. Number of electrons increased in conduction band of SnS_2_ via direct photogenerated electrons as well as releasing electrons by adsorbed oxygen ions after reacting with photoexcited holes (Fig. 17a). As a result, increased electrons in the conduction band of SnS_2_ attracted more number of NO_2_ molecules, and thereby, sensitivity was enhanced through charge transfer. On the other hand, thermal activation first enhances the sensitivity of the sensor with increase temperature from 100 to 110 °C (Fig. 17b) and later sensitivity was severely decreased above 110 °C temperature due to higher desorption rate than adsorption rate. Further, Wu et al. enhanced the gas response of SnS_2_ sensor under light source via deliberately generated nanoscale defects as sulphur vacancy [144]. Sulphur vacancy containing SnS_2_ showed excellent gas response with detection as low as 2.5 ppb at RT under UV illumination. Besides the photoexcited electron–hole pairs, sulphur vacancy acted as additional adsorption sites with high adsorption energy for NO_2_ which was also verified via density functional theory calculations.

To further enhance the sensitivity of the SnS_2_ gas sensor, increased adsorption sites on sensing materials play a crucial role. In this view, Huang et al. demonstrated a RT ultrasensitive ammonia detection under white light irradiation using suspended SnS_2_ layers which are shown in Fig. 17c [145]. The suspended structure increased sensing surface area for more number of NH_3_ molecules interaction and also eliminated charge trap states at SnS_2_/SiO_2_ interface due to existence of air between SnS_2_ and SiO_2_ (substrate). As a result, the suspended SnS_2_ sensor exhibited about three times higher sensitivity to NH_3_ with faster response–recovery rate than that of the traditional SnS_2_ sensor. Moreover, the sensor showed high selectivity towards NH_3_ with detection as low as 20 ppb at RT under white light illumination. The enhancement in sensitivity was attributed to direct charge transfer as well as modulation of Schottky barrier upon exposure to NH_3_, as illustrated in Fig. 17d. In addition, Wang et al. further enhanced the sensitivity of SnS_2_ sensor at RT by synthesizing SnS_2_/rGO nanohybrids [146]. The n-type SnS_2_/rGO nanohybrids exhibited about five times higher sensitivity to 10 ppb NO_2_ with detection as low as 0.15 ppb and also showed a fast response and complete recovery at RT under red light (650 nm) illumination. The enhanced sensitivity was attributed to additional photoexcited electron–hole pairs and modulation of the potential barrier at SnS_2_/rGO interface upon exposure to NO_2_ gas.

### Other Materials

ReS_2_ is a member of VII-group layered TMDCs family with distorted triclinic CdCl_2_-type layer structure contrary to the hexagonal structure of VI-group TMDCs materials [147]. In contrast to VI-group TMDCs materials, VII-group TMDC ReS_2_ possesses an extra d-orbital electron which introduces different and unique properties into ReS_2_. The ReS_2_ has shown great interest in advance electronic devices owing to in-plane anisotropy, interlayer coupling and hard to energy bandgap conversion from indirect to direct. Inspiring from all these significant properties, Jiang et al. investigated the photoelectrical properties of ReS_2_ in the different gas environment under red light (633 nm) source [148]. In this work, they fabricated a sensor from mechanically exfoliated ReS_2_ nanosheet and the sensor exhibited different changes in photocurrent values corresponding to various environments such as O_2_, air, and NH_3_, as shown in Fig. 17f. Two important calculated parameters responsivity (*R*_λ_) and external quantum efficiency (EQE) have higher values in NH_3_ ambient than that of in air or O_2_ ambient. This improvement was due to strong electronic interaction or higher charge transfer between NH_3_ and ReS_2_ which was also verified by first-principles calculations. Adsorbed NH_3_ molecules have better adsorption energy of -205 meV compared to -130 meV of O_2_ on ReS_2_ surface. Physisorption of molecules substantially changed the carrier density of the ReS_2_ through charge transfer (Fig. 17g), and hence, the device showed good response to different gases through changing its current value under red light illumination.

Among the discovery of new 2D materials, MXenes as a new family of 2D materials were first discovered in 2011. MXenes have shown their potential in different applications, including water purification, optoelectronics, energy storage, gas sensing, etc. [149–151]. Mochalin et al. examined the effect of H_2_, air, O_2_ and H_2_O vapour on MXene at RT under a light source illumination [152]. Therefore, visible light energy was not sufficient to produce a considerable change in photocurrent. However, under UV illumination, the MXene exhibited significant photoresponse owing to containing in situ formed phase of TiO_2_ (Fig. 17e). The photoinduced current decay was observed very long time (~ 24 h) in inert ambient due to long relaxation process. Oxygen-containing species such as O_2_ and H_2_O vapour accelerated the relaxation process and achieved fast decay of photoinduced current. This reversible process was obtained due to electron trapping by electronegative atoms as well as intercalation and swelling of MXene which reduced the electrical connection of MXene flakes. Thus, these results lead the utilization of MXene in optoelectronic gas sensor on a commercial platform.

## Conclusion and Outlook

In this comprehensive review, gas sensing characteristics of different materials including metal oxide semiconductors (MOS), and emerging 2D materials, under the light illumination are presented. In the context of MOS materials, photoactivation has been proved to be a promising technique to enhance the gas sensing performances at RT. As discussed, in most cases, photoactivation can be an effective method to replace thermal heating activation to achieve detection of gases at RT. Removing the micro-heater from MOS sensors decreases power consumption and reduces number of fabrication steps and which results in portable and miniaturized gas sensors for emerging IOT applications. Light illumination on MOS improves the generation of photoelectrons and modulates the carrier density in MOS, thus influence on the sensing properties can be expected. Improved sensing response, response–recovery speed and selectivity have been obtained using MOS such as SnO_2_, ZnO and WO_3_ to detect NO_2_, O_3_ and O_2_. Mostly, individual metal oxide sensors exhibit gas sensing at RT in only UV wavelength region due to their higher energy bandgap and exploitation of UV light source over the long time is harmful for human beings. In this regard to further improve the photoactivation, various photosensitizers have been applied to MOS to expand the light absorption spectrum. It is important to note that the LSPR adsorption of noble metals can extend the light absorption spectrum into the visible range. This has driven the research in photoactivated gas sensors from UV to visible lights such as blue, green, and red, as well as the mixed monochromatic, i.e., the white light. Heterojunctions of MOS can improve the separation of photoexcited electron–holes. When designing photoactivated sensors, factors affecting the sensing characteristics to be considered include light intensity, wavelength, size of nanoparticles and film thickness. It also shows that most photoactivated sensors are more sensitive to oxidizing molecules such as NO_2_ and O_2_, although some works reported photo-enhanced sensitivity to H_2_. In the future, more efforts should be explored to develop high-performance detection of organic compounds.

Emerging 2D materials including graphene, TMDCs and MXenes show huge potential in gas sensing at RT under the light sources. Photoactivation enhanced the gas response of 2D materials-based sensors by increasing carrier density through photogenerated electron–hole pairs and also increasing adsorption sites on the surface of 2D materials through desorption of pre-adsorbed atmosphere oxygen ions after reacting with photoexcited holes. Especially, one of the most important problems slow response and recovery kinetics of 2D materials was rectified via photoactivation through improving response time and complete recovery at RT. On the other hand, thermoactivation improves response/recovery kinetics of 2D material sensor; however, it deteriorates sensitivity of the sensor by increasing desorption rate than adsorption rate. Integration of 2D material with other materials improved the sensitivity by including their individual merits and modulation of the potential barrier at the interface upon exposure to gas. Besides the enhanced sensitivity with fast response/recovery kinetics of the sensor, photoactivation improved the reliable selectivity through detection a particular gas by changing the semiconducting behaviour of material from n- to p-type or vice versa and helping to easy movement of carriers at the interface for in a particular direction. For example, 2D g-C_3_N_4_/rGO hybrid showed a response to NO_2_ with its p-type semiconducting behaviour under the dark condition, and in contrast, the hybrid exhibited a response to SO_2_ with its changed n-type behaviour under UV illumination. Despite the high sensing performance of 2D materials heterostructures-based gas sensors, synthesis of large scale and high quality of heterostructures of 2D materials is limited and not yet reached on commercial platforms.

The detection of gases at elevated temperature is one of the major drawbacks of MOS sensors which has been addressed via photoactivation through generating active adsorbed oxygen ions on MOS surface for performing redox reaction with target analytes. On the other hand, emerging 2D materials-based gas sensors show detection of gases at RT through charge transfer mechanism without using any extra stimuli thermal or optical energy sources. However, slow response and incomplete recovery at RT problems of these sensors have been rectified under photoactivation. Besides the UV light, white light is also sufficient for reducing the desorption barrier to ease desorption of gases from sensing 2D materials surface due to the smaller bandgap of 2D materials in the visible range. The sensing results such as sensor response and response/recovery dynamics are not always enhanced. Light activation can sometimes lead to a compromise between the sensitivity and recovery performance of the gas sensor. As a result, selection of light source with proper wavelength and power intensity should be in priority for designing an optoelectronic gas sensor. Besides the perfect selection of appropriate light sources, choosing an individual material or a combination of materials (in the form of nanocomposites and heterostructures) and engineering in sensing material structure would assist to further enhance sensitivity and selectivity of the optoelectronic sensors. The exploitation of van der Waals heterostructures of 2D materials in gas sensing would be a new exciting area in optoelectronic sensor field for multifunctional sensing applications because they have already shown great potential in optoelectronic field owing its remarkable and extraordinary optical properties. The proper selection of 2D materials in van der Waals heterostructure would make selective and highly sensitive sensing platform through significant change in band alignment and carriers transport by the contribution of each and every constituents materials. In addition, 2D materials would also be good candidates for developing optoelectronic gas sensors on flexible and wearable sensing platform for a particular sensing application due to their excellent flexibility and stretchability properties.

Photoactivation has improved gas response with fast response/recovery kinetics at RT, but there is limited research for improving selectivity and stability of the sensor. Moreover, a general consent on the correlations between the light activation, sensor structure and materials selection is still missing. So, optimization of the sensor structure, lighting conditions and initialization of sensor state would be helpful to obtain the best performance. Beyond the laboratory gas sensing results under photoactivation, testing and analysing of the sensor for detecting gas in presence of interfering gases in the environment are challenging task. Generally, an external light source like Xe-lamps or LEDs is used for photoactivation; however, advent of the Internet of things (IOTs), miniaturized sensors with an integrated light source as an appealing monolithic integration of sensing materials on a micro-LED is highly urgent. So, advanced micro-fabrication techniques for implementing innovative sensor designs for lower power consumption, more uniform irradiation of the sensor materials and higher photon energy efficiency should be further studied. Many researches in these contexts are still in progress, and we can expect that photoactivation would be a perfect tool for developing a gas sensor for practical applications.
